# Brain age in multiple sclerosis: a study with deep learning and traditional machine learning

**DOI:** 10.1093/braincomms/fcaf152

**Published:** 2025-04-18

**Authors:** Lars Skattebøl, Gro O Nygaard, Esten H Leonardsen, Tobias Kaufmann, Thomas Moridi, Leszek Stawiarz, Russel Ouellette, Benjamin V Ineichen, Daniel Ferreira, J Sebastian Muehlboeck, Mona K Beyer, Piotr Sowa, Ali Manouchehrinia, Eric Westman, Tomas Olsson, Elisabeth G Celius, Jan Hillert, Ingrid Kockum, Hanne F Harbo, Fredrik Piehl, Tobias Granberg, Lars T Westlye, Einar A Høgestøl

**Affiliations:** Department of Neurology, Oslo University Hospital, Oslo 0450, Norway; Institute of Clinical Medicine, University of Oslo, Oslo 0318, Norway; Department of Neurology, Oslo University Hospital, Oslo 0450, Norway; Department of Psychology, University of Oslo, Oslo 0373, Norway; NORMENT, Division of Mental Health and Addiction, Oslo University Hospital, Oslo 0450, Norway; NORMENT, Division of Mental Health and Addiction, Oslo University Hospital, Oslo 0450, Norway; Tübingen Center for Mental Health, Department of Psychiatry and Psychotherapy, University of Tübingen, Tübingen 72074, Germany; Center of Neurology, Academic Specialist Center, Stockholm Health Services, Stockholm 113 65, Sweden; Department of Clinical Neuroscience, Karolinska Institutet, Stockholm 171 77, Sweden; Department of Clinical Neuroscience, Karolinska Institutet, Stockholm 171 77, Sweden; Department of Clinical Neuroscience, Karolinska Institutet, Stockholm 171 77, Sweden; Department of Neuroradiology, Karolinska University Hospital, Stockholm 171 77, Sweden; Department of Clinical Neuroscience, Karolinska Institutet, Stockholm 171 77, Sweden; Department of Neuroradiology, Karolinska University Hospital, Stockholm 171 77, Sweden; Division of Clinical Geriatrics, Center for Alzheimer Research, Department of Neurobiology, Care Sciences and Society, Karolinska Institutet, Stockholm 171 77, Sweden; Division of Clinical Geriatrics, Center for Alzheimer Research, Department of Neurobiology, Care Sciences and Society, Karolinska Institutet, Stockholm 171 77, Sweden; Institute of Clinical Medicine, University of Oslo, Oslo 0318, Norway; Division of Radiology and Nuclear Medicine, Oslo University Hospital, Oslo 0450, Norway; Division of Radiology and Nuclear Medicine, Oslo University Hospital, Oslo 0450, Norway; Department of Clinical Neuroscience, Karolinska Institutet, Stockholm 171 77, Sweden; Division of Clinical Geriatrics, Center for Alzheimer Research, Department of Neurobiology, Care Sciences and Society, Karolinska Institutet, Stockholm 171 77, Sweden; Department of Neuroimaging, Centre for Neuroimaging Sciences, Institute of Psychiatry, Psychology and Neuroscience, King’s College London, London SE5 8AF, UK; Department of Clinical Neuroscience, Karolinska Institutet, Stockholm 171 77, Sweden; Department of Neurology, Oslo University Hospital, Oslo 0450, Norway; Institute of Clinical Medicine, University of Oslo, Oslo 0318, Norway; Department of Clinical Neuroscience, Karolinska Institutet, Stockholm 171 77, Sweden; Department of Clinical Neuroscience, Karolinska Institutet, Stockholm 171 77, Sweden; Department of Neurology, Oslo University Hospital, Oslo 0450, Norway; Institute of Clinical Medicine, University of Oslo, Oslo 0318, Norway; Center of Neurology, Academic Specialist Center, Stockholm Health Services, Stockholm 113 65, Sweden; Department of Clinical Neuroscience, Karolinska Institutet, Stockholm 171 77, Sweden; Department of Clinical Neuroscience, Karolinska Institutet, Stockholm 171 77, Sweden; Department of Neuroradiology, Karolinska University Hospital, Stockholm 171 77, Sweden; Department of Psychology, University of Oslo, Oslo 0373, Norway; NORMENT, Division of Mental Health and Addiction, Oslo University Hospital, Oslo 0450, Norway; K.G. Jebsen Center for Neurodevelopmental Disorders, University of Oslo, Oslo 5832, Norway; Department of Neurology, Oslo University Hospital, Oslo 0450, Norway; Institute of Clinical Medicine, University of Oslo, Oslo 0318, Norway; Department of Psychology, University of Oslo, Oslo 0373, Norway

**Keywords:** brain age gap, scanner variability, artificial intelligence, neurodegeneration, magnetic resonance imaging

## Abstract

‘Brain age’ is a numerical estimate of the biological age of the brain and an overall effort to measure neurodegeneration, regardless of disease type. In multiple sclerosis, accelerated brain ageing has been linked to disability accrual. Artificial intelligence has emerged as a promising tool for the assessment and quantification of the impact of neurodegenerative diseases. Despite the existence of numerous AI models, there is a noticeable lack of comparative imaging data for traditional machine learning versus deep learning in conditions such as multiple sclerosis. A retrospective observational study was initiated to analyse clinical and MRI data (4584 MRIs) from various scanners in a large longitudinal cohort (*n* = 1516) of people with multiple sclerosis collected from two institutions (Karolinska Institute and Oslo University Hospital) using a uniform data post-processing pipeline. We conducted a comparative assessment of brain age using a deep learning simple fully convolutional network and a well-established traditional machine learning model. This study was primarily aimed to validate the deep learning brain age model in multiple sclerosis. The correlation between estimated brain age and chronological age was stronger for the deep learning estimates (*r* = 0.90, *P* < 0.001) than the traditional machine learning estimates (*r* = 0.75, *P* < 0.001). An increase in brain age was significantly associated with higher expanded disability status scale scores (traditional machine learning: *t* = 5.3, *P* < 0.001; deep learning: *t* = 3.7, *P* < 0.001) and longer disease duration (traditional machine learning: *t* = 6.5, *P* < 0.001; deep learning: *t* = 5.8, *P* < 0.001). No significant inter-model difference in clinical correlation or effect measure was found, but significant differences for traditional machine learning-derived brain age estimates were found between several scanners. Our study suggests that the deep learning-derived brain age is significantly associated with clinical disability, performed equally well to the traditional machine learning-derived brain age measures, and may counteract scanner variability.

## Teaser

Skattebøl *et al*. report on a comparison of deep learning and traditional machine learning brain age estimation in multiple sclerosis using structural MRIs. The deep learning model correlated strongly with chronological age and disability, suggesting that it can provide reliable brain age estimates. Both models performed similarly, but deep learning countered scanner variability.

## Graphical Abstract

**Graphical Abstract:**
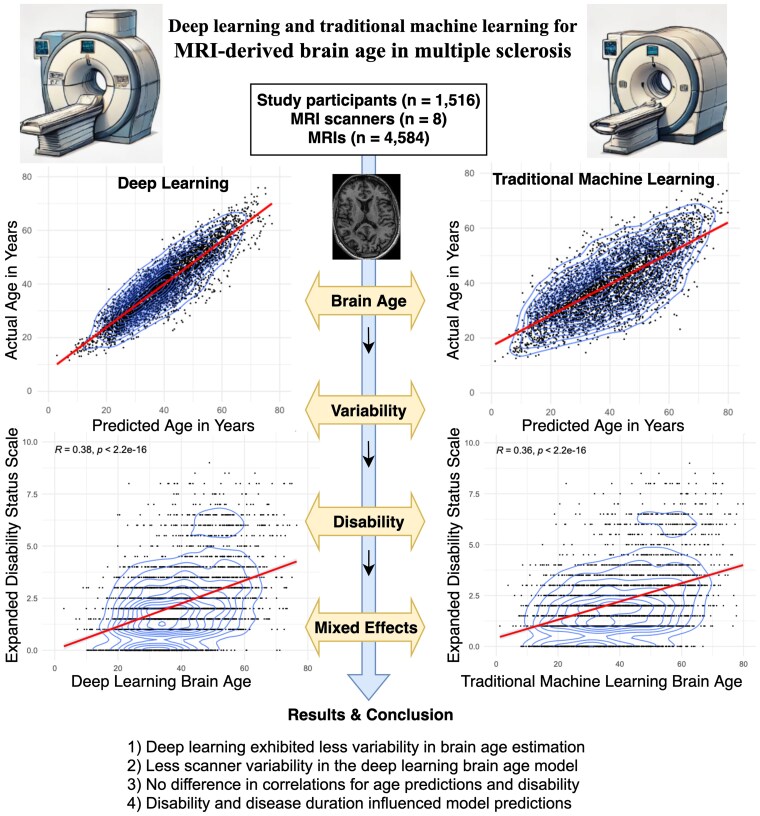

## Introduction

Advancements in immunomodulatory treatment for people with multiple sclerosis (pwMS) have reduced relapse rates and inflammatory lesion activity on MRI.^1^ Nevertheless, some pwMS experience progression independent of relapse activity (PIRA), highlighting the need for additional markers of disease activity.^2-4^ Conventional visual assessment of MRI remains the standard of care for diagnosis and monitoring of multiple sclerosis (MS); however, effective tools for quantifying the underlying neurodegenerative aspects of the disease are still lacking. To address this, we evaluated two advanced methods for capturing neurodegenerative patterns and their potential to estimate brain age in MS.

Over the past decades, numerous calculators for estimating biological ageing have been developed, using a wide variety of data, ranging from heart rate variability to DNA methylation.^5^ The neuroimaging-based biomarker, brain age, provides an estimated biological age of the brain by leveraging a combination of structural, functional or other imaging modalities. Brain age estimation is achieved using machine learning (ML) models, including deep learning (DL) and traditional ML, and trained on large brain MRI datasets, thereby integrating patterns associated with ageing.^6^ In a clinical setting, brain age can be used to measure deviations from normal ageing, providing an individualized quantitative measure of neurodegenerative temporal changes in the brain.^7^ In MS, neurodegeneration plays a key role in disability accrual and represents an important cause of PIRA.^8-11^ Several studies have linked brain age with disease duration, progression, long-term outcome in MS, and mortality in healthy controls (HCs).^7,9,12,13^ As such, a potential future application of brain age pertains to disease staging, enabling clinicians to enhance prognostication and personalize treatment selection, even at an early stage.

Neurodegeneration, the primary focus of brain age studies, arises from various inter-related disease mechanisms, such as ionic imbalance, metabolic dysfunction and failure of compensatory mechanisms or remyelination.^14^ Inflammatory-induced pathological mechanisms ultimately culminate in neuroaxonal loss, a hallmark of disease progression in MS. Brain age models are particularly well-suited for assessing the overall neurodegenerative burden by integrating a wide range of input parameters to capture the complexity of such processes.^3,8,9^ Additionally, the natural ageing process influences the progression of these pathological drivers, creating a dynamic interplay between accumulated damage and brain reserve capacity.^15^

DL, a subtype of ML, is able to learn without predefined feature parameters and handles input differently from traditional ML. Unlike DL, traditional ML requires feature engineering, a process where descriptive MRI features are manually selected and combined to form inputs to the modelling process.^16^ For imaging data, DL is implemented as a layered architecture that typically incorporates 2D or 3D convolutions possessing adaptable filters with notable advantages over traditional ML in broad image processing capabilities.^17^ Consequently, DL holds considerable promise as a robust approach for analysing extensive and diverse imaging data.

The brain age paradigm has emerged as a valuable surrogate marker of neurodegeneration across several neurological conditions, including MS, where it plays an important role in quantifying disease burden and tracking disability progression over time.^6,18^ Early brain age models, such as the work by Franke *et al*., utilized relevance vector machine regression, which outperformed earlier support vector machine approaches now considered outdated.^19,20^ Traditional ML models, including Kaufman *et al*.’s XGBoost-based ML-1118 (machine learning 1118 features) and Cole *et al*.’s BrainageR, relies on structural imaging tools like FreeSurfer and statistical parametric mapping (SPM).^21,22^ These models depend on volumetric measures and are constrained by predefined feature extraction.^21,23^ Recent advances in artificial intelligence have facilitated the development of DL models that bypass the limitations of manual feature engineering, enabling them to extract a more comprehensive range of features, capturing subtle structural, functional, and other imaging patterns.^17,24^ The shift from feature-engineered ML models to DL approaches has broadened the scope and improved the precision of brain age estimation, particularly in terms of reducing the mean absolute error (MAE). DL models often demonstrate superior performance in handling the complexity of large imaging datasets, offering a more nuanced understanding of the neurodegenerative processes.

In this study, we examined the practicality of utilizing a DL model for the purpose of investigating brain age in pwMS. We performed a comparative evaluation of brain age using a DL simple fully convolutional network (DL-SFCN) and a well-established ML model (ML-1118) based on regions of interest obtained from FreeSurfer and the Glasser atlas. The main aim of this study was to validate the DL-SFCN brain age model. Our primary objective was to examine the clinical correlation between brain age and the reliability of the DL-SFCN at different stages of the disease, using the ML-1118 model as a reference. Furthermore, we also sought to assess the performance of the DL-SFCN under different field strengths and across various MRI scanners.

## Materials and methods

### Dataset

**Figure 1:**
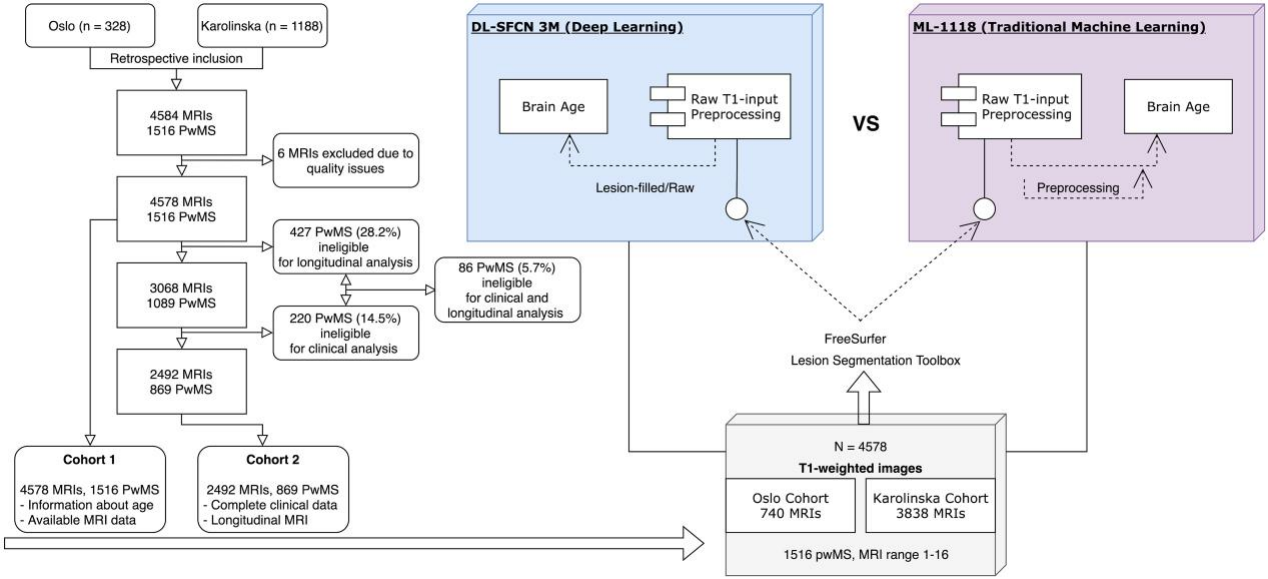
**Standardized MRI data processing for brain age estimation in MS.** To maintain consistency, a standardized pipeline for MRI data was employed for all scans. The dataset was composed of 4584 structural T_1_-weighted MRIs, of which 4578 were utilized for DL, obtained from 1516 individuals with MS from Oslo (746 scans) and Karolinska (3838 scans). Preprocessing of the imaging data involved the use of FreeSurfer and LST. Additionally, an auxiliary preprocessing step was necessary for ML-1118. Both models utilized raw T_1_-MP-RAGE images as input for brain age estimation. Abbreviations: pwMS, people with multiple sclerosis; DL-SFCN, deep learning simple fully convolutional network; ML-1118, machine learning 1118 features.

We conducted a retrospective observational study on a large longitudinal cohort of pwMS diagnosed between 1976 and 2020. MRI brain scans were obtained between 2001 and 2020. Study participants diagnosed in 2001 or later were in accordance with the McDonald's criteria.^25^ Longitudinal MRI data were collected from eight different scanners and consolidated into one large cohort derived from the Karolinska University Hospital (1188 pwMS) and Oslo University Hospital (328 pwMS). Our analysis included a total of 4584 MRIs from 1516 pwMS together with 1199 MRIs from 862 HCs (females: 56.1%, mean age 48.4 years, age range 18.1–94.7 years) as a reference. The majority of pwMS had follow-up according to national guidelines, including an Expanded Disability Status Scale (EDSS) score acquired within 6 months of the MRI.^26^ Clinical and demographic data, including age, sex, date of diagnosis, disease phenotype, disease-modifying therapy (DMT) and EDSS scores, were obtained from National MS registries (Table 1). High-efficacy DMTs included fingolimod, natalizumab, alemtuzumab, rituximab, ocrelizumab, cladribine and mitoxantrone. Six MRI scans were excluded from the DL-SFCN data due to low-quality scans, leaving 4578 scans for the DL brain age analyses. Figure 1 provides a study overview with the cohort selection and pipeline design.

**Table 1:** Baseline clinical values and characteristics at first MRI

Characteristics		Cohort		
	Overall (*n* = 1516)	Karolinska (*n* = 1188)	Oslo (*n* = 328)	Cohort comparison *P*-value
Females^a^	1099 [72.5]	862 [72.6]	237 [72.3]	0.914
Age^b^	38.9 [11.6, 11.6–75.8]	38.9 [11.9, 11.6–75.8]	38.7 [10.3, 18.5–68.3]	0.725
Disease duration^b^	5.6 [7.2, −3.12–50.4]	5.7 [7.4, 0–50.4]	5.1 [6.5, −3.12–36.9]	0.168
EDSS^c^	2.0 [1.0–3.0]	2.0 [1.0–3.0]	2.0 [1.0–3.0]	0.532
EDSS category^a,d^	-	-	*-*	-
≤3.5	1065 [79.8]	769 [87.2]	296 [90.2]	0.125
4.0–6.0	110 [7.2]	84 [9.5]	26 [7.9]	0.373
>6.0	35 [2.3]	29 [3.3]	6 [1.8]	0.127
DMT class^a^	-	-	-	-
None	908 [59.9]	777 [65.4]	131 [39.9]	<0.001
Effective	474 [31.3]	365 [30.7]	109 [33.2]	0.392
Highly effective	127 [8.4]	39 [3.3]	88 [26.8]	<0.001

Baseline clinical values and characteristics for the entire study population at first visit from Karolinska (*n* = 1188) and Oslo (*n* = 328). *P*-values, indicating statistical significance of two-tailed Welch's *t*-test for mean group differences (Karolinska and Oslo) where applicable.

EDSS, expanded disability status scale; DMT, Disease-modifying treatment.

^a^Number [and percentage of total study participants].

^b^Mean years [standard deviation and range].

^c^Median [interquartile range].

^d^Missing data: percentage available data.

### MRI acquisitions

All study participants were scanned at least once on a 1.5 Tesla (3311 MRIs, 72.2%) or 3 Tesla scanner (1273 MRIs, 27.8%). The majority of the entire MS cohort underwent scanning at least twice (*n* = 1,089, 71.8%) and up to a maximum of 16 times (median = 2). The imaging protocol included standardized 3D T_1_-weighted imaging and 2D or 3D T_2_-weighted fluid-attenuated inversion recovery (FLAIR). For our HCs, MRI was conducted at time points spanning up to 18 times (median = 1). The various MRI acquisition parameters can be found in Supplementary Table 1.

### MRI analyses

The DL and ML algorithms were both employed to directly estimate brain age from T_1_-weighted magnetization prepared rapid gradient-echo (MP-RAGE) images. Preprocessing of the data varied by site; at Karolinska, cortical reconstruction and volumetric segmentation were performed using FreeSurfer version 5.3.0, while in Oslo, FreeSurfer version 7.1.0 was used. To address potential variability introduced by different FreeSurfer versions, a separate external subset analysis on MRIs (*n* = 81) derived from a separate local MS cohort using MP-RAGE on the same 3 T scanner was used to compare predicted brain age outcomes from the two versions. In a subset of the study sample (734 MRIs, Oslo cohort), lesions were segmented using a lesion prediction algorithm implemented in Lesion Segmentation Toolbox (LST) version 3.0.0 for statistical parameter mapping (www.statistical-modelling.de/lst.html) on FLAIR images.^27^ Lesions filled and non-lesions filled were compared to address a possible induced volumetric bias.

### Brain age estimation models

**Figure 2:**
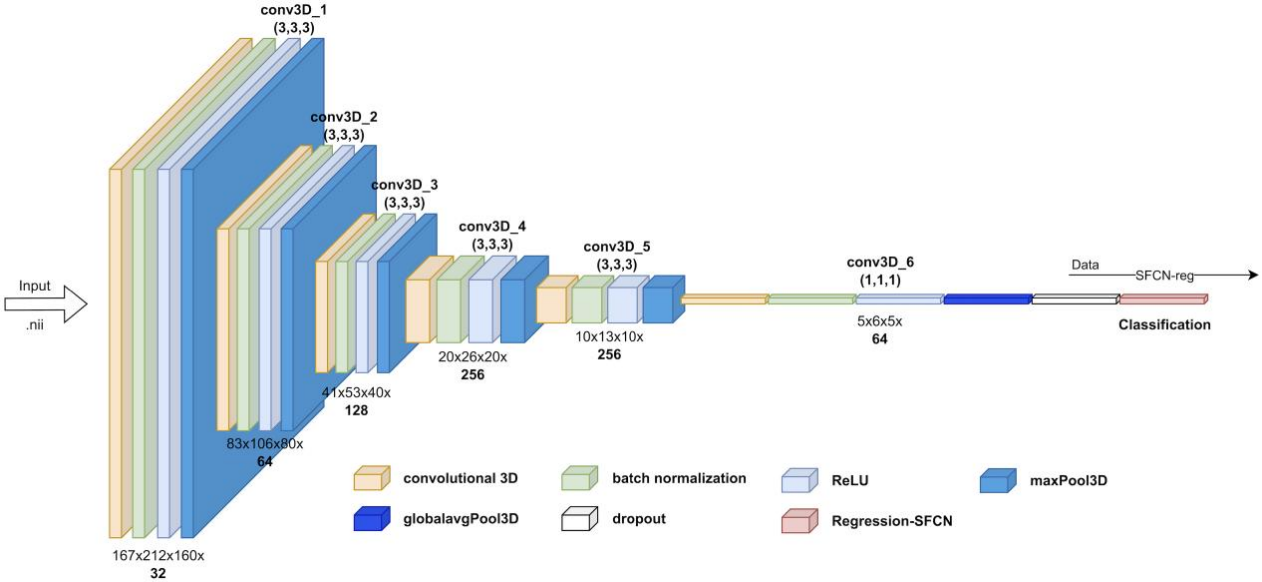
**Architecture of regression 3D SFCN deep learning model.** The architecture of the regression 3D SFCN deep learning model, designed for 3D regression tasks, is composed of several layers and operations, each processing the input data. The conv3D in each block applies convolutional operations, which are mathematical processes that filter the input data using learnable filters or kernels applied to 3D input data (e.g. volumes or 3D images). Batch normalization is used to normalize the input to each layer by adjusting and scaling activations, helping to stabilize and speed up the training process by reducing internal covariate shift. The ReLU serves as the activation function, introducing non-linearity by setting negative values to zero and determining whether a neuron will propagate to the next layer. GlobalAveragePooling3D reduces the spatial dimensions of the 3D feature map by calculating the average value of each feature across the entire spatial volume, producing a single value per feature channel. The final block includes a dropout layer, a regularization technique used during training to prevent overfitting by randomly setting a fraction of the input units to zero at each update, followed by the application of the SFCN-reg (simple fully convolutional regression) function, which predicts continuous values (e.g. *ŷ_i_*—Predicted brain age for subject *i*). The model utilizes the neuroimaging file format ‘.nii’ (neuroimaging informatics technology initiative) for storing neuroimaging data such as MRI scans. Abbreviations: conv3D, convolution 3D; ReLU, rectified linear unit; SFCN-reg, simple fully convolution regression; ‘.nii’, neuroimaging informatics technology initiative.

The study utilized a pretrained DL model trained on HCs over 3 years of age (52% females, age range 3–95 years) derived from 21 datasets.^28^ The simple convolutional neural network (SFCN) architecture (Supplementary Table 2), optimized by Leonardsen *et al*. through mean squared error minimization, consists of consecutive interconnected layers. These include 3D convolutions (conv3D), batch normalization, rectified linear activation functions (ReLU), max pooling (maxPool3D), global average pooling (globalavgPool3D), a dropout layer and a final classification layer (Fig. 2). Peng *et al*. introduced soft labelling in the original DL-SFCN model to address uncertainty in age prediction, using a Kullback–Leibler divergence loss to estimate probability distributions rather than single values; however, Leonardsen *et al*. demonstrated that hard labels provided more precise and reliable brain age predictions across diverse datasets, proving better suited for capturing the complex relationship between biological age and brain health.^28,29^ In preparation for this study, a reoptimization was performed by testing different classifier activation functions, in which the DL-SFCN regression classifier (SFCN-reg) outperformed SoftMax classification and ranking-based SFCN models in terms of achieving a lower MAE at 3.90 and *R*^2^ = 0.94 on an external dataset with varying scanners and field strengths.^28^ SFCN-reg predicts a single value for each participant, serving as a direct estimated value for the brain age, and we deemed it to be the best choice due to the varying nature of our dataset. The pretrained model used convolutional layers and had 3 million feature parameters.

The ML-1118 model consisted of a pretrained regularizing gradient boost framework (https://github.com/tobias-kaufmann/brainage), specifically utilizing eXtreme Gradient Boosting (XGBoost) implemented in the R programming language, with an MAE of 5.26 years and *R*^2^ = 0.82 on our test data (*n* = 862). ML-1118 was trained using 35 474 HCs, of which 54% were females, with an age range of 3–89 years, and built upon a traditional ML substructure and engineered to handle 1118 feature parameters.^21^ These features were extracted from parcellated regions of interest in the cortex, subcortex and cerebellum using FreeSurfer and the Glasser atlas/Human Connectome Project.^30^

The training data for the pretrained brain age models consisted exclusively of cross-sectional MRI scans from unique HCs. For the MS cohort, longitudinal MRI data facilitated stronger correlations with clinical measures such as EDSS and disease duration, likely due to its ability to capture disease progression over time, an advantage not fully reflected in single time-point analyses.

We evaluated brain age in all pwMS, accounting for all brain regions, and engaged both models to calculate and compare the brain age gap (BAG) to reduce the chronological age-related effect on EDSS.


$$\mathrm{BA}G_{i}\;=\;({\hat{y}}_{i}\;-\;(\beta_{o}+\;\beta_{1}y_{i}+\beta_{2}y_{i}^{2}+\beta_{3}S_{i}+\beta_{4}G_{i})\;-\;y_{i})$$

where ${\hat{y}}_{i}$ is the predicted brain age for subject *i*, *y_i_* is the chronological age for subject *i*, $\beta_{o}$ is the intercept from regression mode l, $\beta_{1}y_{i}$ is the linear effect of chronological age, $\beta_{2}y_{i}^{2}$ is the quadratic effect of chronological age squared for non-linear relationships, $\beta_{3}S_{i}$ is the effect of scanner type, where *S_i_* represents the scanner variable for subject *i* and $\beta_{4}G_{i}$ is the effect of sex, where *G_i_* is a binary variable representing the subject’s sex.

BAG denotes the incongruity between chronological age and biological age, with the extent of deviation from chronological age being symbolized as Δ age, or the variance between the predicted ‘Age’ and the chronological ‘Age’. We employed BAG to reduce the chronological age-related effect on EDSS.

### Statistical analysis

The statistical analyses were conducted in R version 4.1.1, with adjustments made for age, age^2^, sex and scanner by regression of the residuals in the brain age estimates.^31,32^ Stratification by field strength and EDSS category was used for pairwise comparisons and sub-analyses.

Pearson's correlation coefficient was used for correlation analyses, with a significance level set at <0.05 using regular linear regression models. We used Spearman's rank correlation for non-linear, ordinal scale and not normally distributed data, e.g. EDSS. A Bonferroni correction was used for pairwise comparisons of parametric data. Two-sample Welch's or Pooled *t*-tests were used to compare mean values when unequal or equal variances were assumed as indicated by a significant *F*-test. A non-parametric approach pertaining to the comparison of median EDSS scores was used to compare the levels of clinical severity and Chi Square Kruskal–Wallis test for multiple comparisons of non-parametric scanner-level data with unequal variances. Levene’s test was used to assess scanner variability (Brain age and BAG variability) for inter- and intra-scanner-derived estimates. Cohen's *D* was employed to evaluate effect sizes. Effect sizes exceeding 0.2, 0.5 or 0.8 were construed as indicative of small, medium or large effects, respectively.

The associations between brain age and clinical variables in longitudinal data were investigated using linear mixed effects (LME) modelling, with the lme4 and robustlmm packages in R, and restricted maximum likelihood set to false. The fixed effects were EDSS, disease duration, age, sex, phenotype, DMT class and scanners, while random effects were subject identifier and site to ensure variability handling at site and subject levels, avoiding overestimating the fixed effects. Phenotype, gender, DMT class and scanner were encoded as categorical variables internally in R. Each category got a separate coefficient, with one category used as the reference level. Brain age and BAG from ML-1118 and DL-SFCN were set as response variables for comparison of model outcome.

An intraclass correlation coefficient (ICC) was used to assess the reliability of DL-derived brain age estimates from lesion-filled and non-lesion-filled data. We employed a one-way random-effects model (ICC(1,1), Koo and Li (2016) classification) to evaluate single-measurement consistency, using the ‘ICC’ package in R. This model assumes that the raters (lesion- and non-lesion-filled data) were randomly drawn from a larger population, making it suitable for assessing consistency across different preprocessing conditions.^33,34^

An *F*-test was employed to assess the ratio of variances and to compare the squared difference between each brain age estimate and the mean brain age. Brain age estimates and BAG did not follow a normal distribution on an individual scanner level, measured visually with a Q plot and histogram, and confirmed quantitatively by the Shapiro–Wilk test.

A robust LME model was explored to correct for potential outliers, with three robust LME iterations calculated and evaluated for optimal model fitness, differing in tuning parameters for the Huber p-function and iterative robustness and efficiency, using the same independent variable set as in the classic LME (Supplementary Fig. 1).

We tested for a statistical difference between DL- and ML-derived correlations for BAG output with respect to EDSS using the ‘cocor’ package in R. Supplementary Table 3 provides further details on the approach and outcomes.

### Ethical approval and reporting guidelines

The study received approval from the Regional Ethics Review Board in Stockholm (ID: 2009/2107-31/2, 2018/2711-32), the Institutional Review Board (IRB) at Huddinge Hospital, Stockholm, Sweden (ID: 21/95) and the Regional Committees for Medical and Health Research Ethics (REK) at Oslo University Hospital, Norway (ID: 2011/1846 A and 2016/102). All participants were anonymized and provided consent by signing a consent form prior to participating in the study. The study was conducted in accordance with the guidelines of the Declaration of Helsinki.

### Role of funding

The study design, data collection and analysis, decision to publish and manuscript preparation were not influenced by the funder.

## Results

### Demographics and patient characteristics

Our study cohort included 1516 pwMS, with a mean age of 38.9 years, a median EDSS score of 2.0 (with an interquartile range of 1.0–3.0) and a disease duration of 5.6 ± 7.2 years at the baseline. The majority of pwMS had relapsing-remitting MS (*n* = 1,180, 78%). In Oslo, some MRIs were obtained prior to diagnosis (*n* = 20; mean 0.4 years), resulting in a negative range value for disease duration (range −3.12 to 50.4 years). However, the effect of this is negligible. Baseline demographic and clinical characteristics can be found in Table 1.

There were no significant differences in sex distribution, age, disease duration or EDSS by site comparison at study entry. A difference was found in the use of high-efficacy DMTs (Karolinska: 3.3%, Oslo: 26.8%, *P* < 0.001) and no treatment status for pwMS (Karolinska: 65.4%, Oslo: 39.9%, *P* < 0.001) between the sites. The average time to reach high-efficacy treatment, whether transitioning from effective treatment or no treatment, was 3.61 years into follow-up. EDSS scores were absent for 20.2% of the cases, primarily attributable to the absence of EDSS records in the National registries during the period encompassing the MRI scans.

Overall, 59.9% (*n* = 908) was not undergoing any treatment at the time of the initial MRI scan, while 70.3% displayed an EDSS score of ≤3.5. For pwMS on effective or high-efficacy DMTs, EDSS scores were available for the majority of participants (90.7 and 96.9%, respectively). Using data from multiple time points, our analyses revealed that pwMS exhibiting primary progressive MS (PPMS, *n* = 206 [14.9%], 66.5% females) had a significantly higher EDSS score than those with relapsing-remitting MS (RRMS, *n* = 1180 [85.1%], 73.7% females; *P* < 0.001). Mean age in the PPMS group was on average 11 years higher than in the RRMS group (*P* < 0.001). Conversion from RRMS to SPMS occurred in 8.2% (*n* = 89) and on average after 9.1 years of follow-up.

### Brain age and chronological age

**Figure 3:**
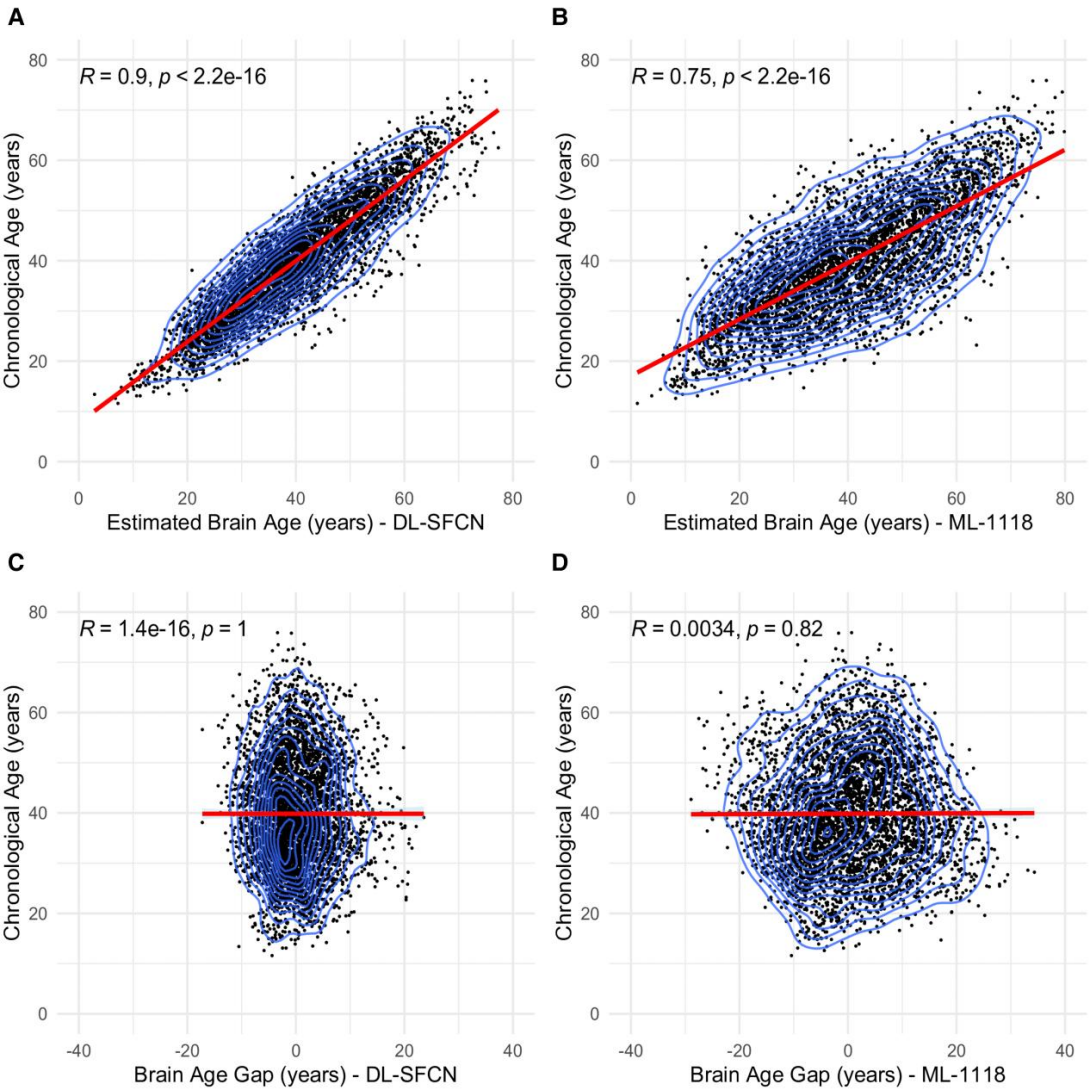
**Correlation between chronological age and brain age estimation using DL-SFCN and ML-1118 models in longitudinal data.** This figure presents scatter plots with layered density outlines (Panels **A–D**) depicting the relationship between chronological age and brain age estimation in years, as derived from two models, DL-SFCN and ML-1118, using longitudinal data. Panels **A** and **B** illustrate the correlations between chronological age and estimated brain age for DL-SFCN (Panel **A**: *R* = 0.9, *P* < 0.001, *n* = 4584) and ML-1118 (Panel **B**: *R* = 0.75, *P* < 0.001, *n* = 4584), respectively. Panels **C** and **D** show the correlation between chronological age and BAG derived from DL-SFCN (Panel **C**: *R* < 0.001, *P* = 1) and ML-1118 (Panel **D**: *R* = 0.003, *P* = 0.82). The brain age estimations were residualized to account for variations in mean predicted values for age, age², scanner and gender. Abbreviations: *R*, Pearson’s correlation coefficient; *P*, *P*-value; DL-SFCN, deep learning simple fully convolutional network; ML-1118, machine learning 1118 features; BAG, brain age gap.

The DL-SFCN brain age estimates demonstrated a correlation of 0.90 with chronological age (Fig. 3A) (*R* = 0.85 unadjusted; Supplementary Fig. 2A), whereas the ML-1118 brain age exhibited a correlation of 0.75 (Fig. 3B) (*R* = 0.64 unadjusted; Supplementary Fig. 2B). After bias correcting for age, BAG did not correlate with chronological age using DL-SFCN (*R* < 0.001, *P* = 1; Fig. 3C) or ML-1118 (*R* = 0.003, *P* = 0.82; Fig. 3D). However, for the ML-1118, using its corresponding standard deviation as plot range, a positive correlation with chronological age was found (*R* = 0.16, *P* < 0.001), and a negative correlation for all positive numbers (*R* = −0.17, *P* < 0.001), suggesting a possible sigmoidal offset. This effect was not apparent in the DL-SFCN. The unadjusted age-BAG correlates can be found in Supplementary Fig. 2C and D.

A cross-sectional comparison of baseline MRIs revealed a difference in BAG between pwMS and HCs, wherein pwMS exhibited 3.4-year higher BAG as compared with HCs (*P* < 0.001). Chronological age (*t* = 22.17; *r* = 0.34, *P* < 0.001) and disease duration (*t* = 37.65; *r* = 0.49, *P* < 0.001) correlated significantly with EDSS. The inter-model variability for both brain age and the BAG metric in decade-wise age groups was significant at all levels, except for pwMS aged 71–80 years, with DL-SFCN demonstrating significantly lower variability compared with ML-1118 across most age groups, indicating more stable and precise brain age predictions (Supplementary Table 4).

We performed a sub-analysis (*n* = 81) to test for variance in brain age between FreeSurfer 5.3.0 and 7.1.0. We found that the variance was higher for FreeSurfer 5.3.0 (*F* = 1.60, *P* = 0.036), and the intercept for FreeSurfer 5.3.0 was statistically significant (7.837, *P* = 0.0247), whereas the intercept for 7.1.0 was not (−1.421, *P* = 0.754). Both versions had similar *R*-squared values (*R*^2^_5.3.0_ = 0.525, *R*^2^_7.1.0_ = 0.512) and there was no significant difference in correlation between chronological age and predicted brain age (Age versus pAge_5.3.0_, *r* = 0.725 ∣ Age versus pAge_7.1.0_, *r* = 0.715, *P* = 0.906).

For DL-SFCN, all data were preprocessed using FreeSurfer 7.1.0 (autorecon1) prior to brain age estimation.

DL brain age estimates from a subset of lesion-filled and non-lesion-filled data were highly correlated (*r* = 0.99; Supplementary Fig. 3). The test–retest reliability index was excellent (ICC = 0.98). However, a significant difference in the DL-derived brain age for lesion-filled (mean = 40.38 years, Sd = 10.8) and non-lesion-filled data (mean = 41.21 years, Sd = 11.0) was found (*t*(733) = 11.95, *P* < 0.001).

The ratio of inter-model variance (*F*), representing statistical dispersion (Supplementary Table 5), was significant and indicated a 1.4-fold higher variability in ML-1118, compared with DL-SFCN, for longitudinal brain age (*F* = 1.43, 95% CI [1.35, 1.52], *P* < 0.001). This observation remained consistent at EDSS ≤3.5 (*F* = 1.47, 95% CI [1.37, 1.58], *P* < 0.001) and 4.0–6.0 (*F* = 1.33, 95% CI [1.10, 1.61], *P* = 0.003), but insignificant for brain age at EDSS >6.0 (*F* = 1.25, 95% CI [0.89, 1.75]). For BAG, the ratio of variance was significantly different across all EDSS levels. The variability metric for brain age and BAG consistently showed higher values for ML-1118 across all EDSS categories.

### Associations between brain age and clinical variables

The LME results for ML-1118 (Table 2) showed that an increasing brain age was associated with disease duration (*β*1 = 0.22; *t* = 6.51, *P* < 0.001) and EDSS (*β*1 = 0.33; *t* = 4.15, *P* < 0.001). Similarly with DL-SFCN (Table 3), an association between brain age and disease duration (*β*1 = 0.12; *t* = 6.06, *P* < 0.001) and EDSS (*β*1 = 0.27; *t* = 5.34, *P* < 0.001) were found. Uncorrected ML-1118- and DL-SFCN-derived brain age yielded comparable LME results to brain age and BAG adjusted for age, age2, scanner and sex (Supplementary Tables 6–9). In both models, chronological age had the largest effect on brain age estimates (*P* < 0.001).

**Table 2:** Predictors and variability in brain age estimates: ML-1118

ML-1118				
Predictor	Estimates	CI	Statistic	*P*-value
(Intercept)	6.98	0.77–14.73	1.76	0.078
Disease duration	0.22	0.15–0.28	6.45	<0.001**
EDSS	0.33	0.16–0.51	3.70	<0.001**
DMT class	−0.59	−1.12–−0.07	−2.21	0.027**
No treatment	−0.64	−1.05–−0.22	−3.00	0.003**
PPMS	−3.10	−10.66–4.46	−0.80	0.420
RRMS	−3.97	−11.48–3.54	−1.04	0.300
Sex	0.57	−0.52–1.66	1.03	0.300
Age	0.89	0.84–0.94	35.14	<0.001**
*Scanner*	-	-	-	-
Aera	−0.15	−0.69–0.40	−0.52	0.600
Avanto [2]	−0.44	−0.93–0.05	−1.77	0.077
GE 750	3.06	1.83–4.29	4.87	<0.001**
GE Premier	2.50	0.45–4.55	2.40	0.017**
Skyra	1.27	−1.57–4.12	0.88	0.380
Avanto	0.55	−0.91–2.00	0.74	0.460
Vision	−0.91	−1.62–−0.19	−2.49	0.013**

Random effects	
*σ* ^2^	15.87
*τ*_00 (ID Simple)_	71.64
	0.00_Site_
*N*	2_Site_
	1299_ID_Simple_
Observations	3688
Marginal *R*^2^	0.884
Conditional *R*^2^	NA

The outcomes of our linear mixed effects analysis for ML-1118 and the impact of various predictor variables. The ‘Estimate’ column offers estimated coefficients (*β*), indicating the strength and direction of relationships with brain age, along with corresponding *P*-values set at a threshold of 0.05. Siemens Trio was set as the reference level for scanners.

CI, confidence interval; Statistic, *t*-statistic; DMT, disease-modifying therapy; PPMS, primary progressive multiple sclerosis; RRMS, relapsing-remitting multiple sclerosis; *σ*^2^ (sigma-squared), the variance of the individual observations within the same group or cluster; *τ*00 (tau-double-zero), the variance of the random intercepts across different groups or clusters; Marginal *R*^2^, provides the variance explained only by fixed effects.

**Table 3:** Predictors and variability in brain age estimates: DL-SFCN

DL-SFCN				
Predictor	Estimates	CI	Statistic	*P*-value
(Intercept)	0.63	−3.73–4.99	0.28	0.780
Disease duration	0.11	0.08–0.15	5.84	<0.001**
EDSS	0.27	0.17–0.37	5.33	<0.001**
DMT class	−0.20	−0.49–0.10	−1.32	0.190
No treatment	−0.42	−0.65–−0.19	−3.62	<0.001**
PPMS	0.95	−3.30–5.19	0.44	0.660
RRMS	0.34	−3.88–4.55	0.16	0.880
Sex	−0.26	−0.89–0.38	−0.80	0.430
Age	0.94	0.91–0.97	63.40	<0.001**
*Scanner*	-	-	-	-
Aera	0.51	0.21–0.81	3.30	<0.001**
Avanto [2]	0.14	−0.13–0.41	1.03	0.300
GE 750	0.09	−0.62–0.80	0.24	0.810
GE Premier	2.45	1.30–3.61	4.17	< 0.001**
Skyra	4.03	2.45–5.61	5.00	< 0.001**
Avanto	1.08	0.25–1.92	2.55	0.011**
Vision	−0.14	−0.54–0.25	−0.70	0.480

Random effects	
*σ* ^2^	4.76
*τ*_00 (ID Simple)_	24.73
	0.00_Site_
*N*	2_Site_
	1299_ID_Simple_
Observations	3682
Marginal *R*^2^	0.962
Conditional *R*^2^	NA

The outcomes of our linear mixed effects analysis for ML-1118 and the impact of various predictor variables. The ‘Estimate’ column offers estimated coefficients (*β*), indicating the strength and direction of relationships with brain age, along with corresponding *P*-values set at a threshold of 0.05. Siemens Trio was set as the reference level for scanners.

CI, confidence interval; Statistic, *t*-statistic; DMT, Disease-modifying therapy; PPMS, primary progressive multiple sclerosis; RRMS, relapsing-remitting multiple sclerosis; *σ*^2^ (sigma-squared), the variance of the individual observations within the same group or cluster; *τ*00 (tau-double-zero), the variance of the random intercepts across different groups or clusters; Marginal *R*^2^, provides the variance explained only by fixed effects.

We observed a lower mean chronological age (mean 37.3 years, age range 13.5–67.6) among pwMS on high-efficacy DMTs when compared with untreated individuals (mean 40.8 years, age range 11.6–75.9) (*t* = −8.0952, *P* < 0.001). Despite this age difference, the LME demonstrated a negative effect measure of DMT class on estimated brain age in DL- and ML-derived estimates (Tables 2 and 3). However, we found a negative linear regression coefficient for chronological age (*Age = −0.61*  *×*  *Follow-Up Time + 41.91*), most likely representative of a healthy selection bias.

EDSS correlated significantly with chronological age (Rho = 0.33, *P* < 0.001) and disease duration (*r* = 0.29, *P* < 0.001). The estimated brain age, as determined by both ML (Rho=0.36, *P* < 0.001) and DL (Rho = 0.38, *P* < 0.001), both exhibited a moderate correlation with EDSS. Nonetheless, we noted a gradual weakening of the correlation between brain age and BAG as the EDSS category increased (Supplementary Table 10). An LME analysis for each individual EDSS category (EDSS ≤3.5, 4–6 and >6) was conducted, in which the ML-1118-derived brain age was associated with disease duration (*β*1: *t* = 5.86, *P* < 0.001), but not with EDSS at ≤3.5 (Supplementary Tables 11 and 14). In contrast, the DL-SFCN-derived brain age was associated with both disease duration (*β*1: *t* = 5.04, *P* < 0.001) and EDSS (*β*1: *t* = 2.61, *P* = 0.009) at EDSS ≤3.5. Disease duration showed a moderate positive correlation with brain age for both models (DL: *r* = 0.50, *P* < 0.001; ML: *r* = 0.45, *P* < 0.001), regardless of EDSS category. Furthermore, disease duration had a low positive correlation with overall EDSS (*r* = 0.29, *P* < 0.001). Disease phenotype also exerted an influence on BAG, with significantly higher values observed in the PPMS group for both ML-1118- and DL-SFCN-derived BAG (*P* < 0.001). For details concerning brain age LME results related to EDSS 4–6 and >6, please refer to Supplementary Tables 12, 13, 15 and 16.

We explored the significance of a co-correlation model difference for BAG output with respect to EDSS and disease duration using the ‘cocor’ package in R, but no significant inter-model differences were found. Supplementary Table 3 provides further details on the modelling approach and outcomes.

### Inter-scanner variability and field strength

We conducted a sub-analysis on brain age variability and scanner dissimilarities by stratification of scanner, field strength, EDSS category and model output for both brain age and BAG. In terms of field strength, a slight, but negligible group difference in mean chronological age was observed (Cohen's *D* = 0.065, 95% CI [0.00035, 0.13]) when analysing multiple time-point data (*t* = 1.97, *P* = 0.049). The mean age was approximately 40.0 years (age range 13.1–75.9) for 3 T scanners (1273 MRIs) and 39.3 years (age range 11.6–75.8) for 1.5 T scanners (3311 MRIs). To address this age-related confounder, BAG measurements were employed as compensation.

**Figure 4:**
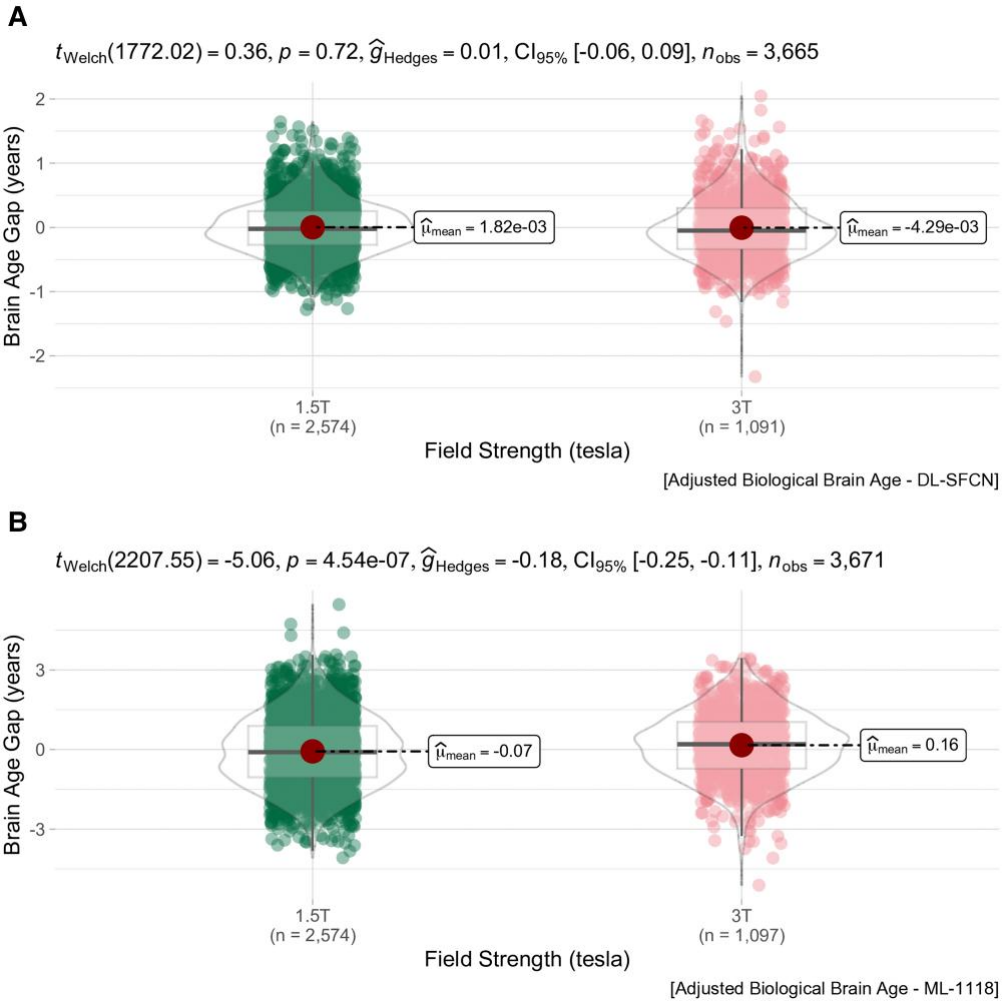
**Comparing BAG estimates at 1.5 T and 3 T Using DL-SFCN and ML-1118 models: a box-violin plot analysis.** The top panel (**A**) represents DL-SFCN-derived BAG estimates, while the bottom panel (**B**) displays ML-1118-derived BAG estimates. Both panels illustrate BAG differences, in years, at 1.5 T (left-sided plots, **A** (*n* = 2574) and **B** (*n* = 2574)) and 3 T (right-sided plots, **A** (*n* = 1091) and **B** (*n* = 1097)) using a box-violin plot. BAG was adjusted via mixed-effects linear regression, controlling for EDSS, disease duration and MS subtype, with Subject ID as a random intercept. Adjusted residuals, representing unexplained variability, were aggregated within each field strength group and visualized using box plots. Pairwise comparisons at both field strengths were conducted using Welch’s *t*-test to account for unequal variance between models. Abbreviations: CI, confidence interval; T, Tesla; *P*, *P*-value.

For field strength, we adjusted for age, gender, EDSS, age at onset, disease duration and subtype and observed a lower median BAG was at 1.5 T compared with 3 T using ML-1118 (95% CI [−0.25, −0.11], *P* < 0.001) (Fig. 4). A field strength effect was not observed with DL-SFCN for BAG (*P* = 0.71). The inter-model BAG was compared by calculating and aggregating the mean residuals for each subject, filtered by field strength, to reduce risk of bias through within-subject variability. A paired *T*-test revealed significant differences for BAG at 3 T (*t* = −4.14, *P* < 0.001) and 1.5 T (*t* = 3.13, *P* = 0.002) between ML-1118 and DL-SFCN. A total of 23.4% had PPMS in the 1.5 T group (573 MRIs), as opposed to 13.4% for 3 T (145 MRIs). However, the median EDSS remained the same, regardless of field strength (1.5 T: median EDSS 2.0, IQR: 1.0–3.0; and for 3 T: median EDSS 2.0, IQR: 1.0–3.0). For scanner variability, the equality of variances between ML-1118 and DL-SFCN were significant by inter- and intra-scanner evaluation, and further suggested that the assumption of homoscedasticity may be violated for BAG in all scanners, except for Siemens Skyra, which could not be accurately calculated due to a low number of observations (Supplementary Table 17). We also examined the inter-scanner difference in BAG measurements and observed a significant distinction between GE 750 and Siemens Aera, Avanto, Trio and Vision when utilizing ML-1118 (*P* < 0.001). Furthermore, at EDSS ≤3.5, the same pattern occurred for ML-1118 (*P* < 0.001). No differences were found for GE Premier. In contrast to ML-1118, the DL-SFCN did not exhibit any apparent scanner-related effects on BAG in multiple time-point data for EDSS ≤3.5 (*n* = 1065) or across all EDSS categories (Supplementary Figs 4–7). However, for both brain age models, and like the strength of correlation between estimated brain age and BAG to EDSS, the scanner effects on BAG dissipated and became insignificant when EDSS was ≥4.0 (*n* = 110) (Supplementary Figs 8–11). Additionally, the LME analysis (Table 2) revealed that GE 750 had a significant effect on the estimated brain age (corrected for age, age2, scanner and sex) from ML-1118. Depending on the reference scanner, GE 750 for ML-1118 (*β*1: 6.77; *t* = 2.97, *P* = 0.003) and DL-SFCN (*β*1: −6.80; *t* = −5.19, *P* < 0.001) revealed a net positive and negative effect on estimated brain age, as indicated in Table 4. In both models, scanner-induced effects on estimated brain age appeared to have a greater impact on EDSS ≤3.5 compared with higher scores (Supplementary Tables 11–16).

**Table 4:** MRI scanner types and their relationship profiles and brain age estimates

Scanner	Participants^a^	MRIs^a^	Age^b^	EDSS^c^	Disease duration^b^	ML brain age^b^	DL brain age^b^
GE Premier	23 (1.5)	49 (1.0)	35.9 (9.4)	1.5 (1.0–2.0)	2.6 (3.2)	35.9 (10.6)	35.9 (10.6)
GE 750	229 (15.1)	491 (10.7)	39.6 (10.1)	2.0 (1.0–2.5)	6.4 (5.9)	43.9 (12.4)	39.6 (12.9)
Siemens Aera	117 (7.7)	749 (16.3)	41.5 (11.3)	2.0 (1.0–3.0)	8.9 (7.0)	41.6 (14.3)	41.5 (11.8)
Siemens Avanto (Oslo)	75 (4.9)	192 (4.2)	37.3 (7.5)	2.0 (1.5–2.5)	2.8 (2.1)	37.7 (11.2)	37.3 (8.5)
Siemens Avanto (Karolinska)	432 (28.5)	1649 (36.0)	40.4 (11.3)	2.0 (1.0–3.0)	8.0 (7.1)	40.5 (14.9)	40.4 (12.2)
Siemens Skyra	1 (0.07)	14 (0.3)	32.3 (−)	2.0 (1.5–2.0)	0.7 (−)	32.2 (9.6)	32.2 (5.1)
Siemens Trio	182 (12.0)	719 (15.7)	39.5 (11.4)	2.0 (1.0–3.0)	7.6 (7.4)	39.9 (15.0	39.5 (13.3)
Siemens vision	457 (30.1)	721 (15.7)	38.6 (11.4)	2.0 (1.0–3.0)	6.3 (7.5)	38.6 (16.8)	38.6 (12.7)

A list of the different MRI scanner types used in the study, the number and percentage of participants, the number and percentage (overall) of MRI scans, the average age of participants with SD, the median EDSS score with IQR, the mean disease duration with SD and the mean traditional ML and DL brain ages (corrected) with their respective SDs. The ‘Siemens Aera’ (1.5 Tesla) scanner contributed the majority of longitudinal MRIs, while ‘Siemens Avanto (Karolinska)’ (1.5 Tesla) produced most of the MRIs.

SD, standard deviation; EDSS, expanded disability status scale; IQR, interquartile range; ML, machine learning; DL, deep learning.

^a^Number [and percentage of total study participants].

^b^Mean years [standard deviation].

^c^Median [interquartile range] learning.

## Discussion

The aim of our study was to investigate and validate a DL-SFCN brain age model, using the traditional ML-1118 as a benchmark. Our results suggest that the utilization of DL neural networks for brain age estimation is a promising and viable approach. The DL model showed a significant correlation and association, in terms of effects measures, with clinical disability and demonstrated less variability at different field strengths with fewer scanner-induced biases compared with ML-1118. The DL-SFCN brain age model was perceived as robust across all scanners and field strengths, as opposed to ML-1118.

### Comparison of DL-SFCN and ML-1118 models

Compared with ML-1118, the DL-SFCN yielded brain age estimates and BAG with lower variability and range. DL-SFCN exhibited a slightly stronger correlation between estimated and chronological brain age compared with ML-1118. MRI-derived measures like white matter and rim-enhancing lesions have been validated in pathological studies, whereas brain age lacks true pathological correlates, making it challenging to determine the extent of over- or underestimation due to the absence of an absolute, biologically verifiable measure of brain age, particularly in pathological cases.^3,35,36^ One potential reason for the precision of DL-SFCN may be its ability to handle data from various scanners and manage extraneous noise better than ML-1118. However, little is known about the precise intra-layer transfer of information in DL or the causal effects of confounders impacting model accuracy. Dispersion issues in artificial intelligence are common, arising from diverse data acquisition like MRI scanners, dimensionality reduction complexities, regression variations impacting model stability, intricate ML architecture compatibility and prediction biases, may result in challenges like overfitting, underfitting, nodal death and gradient errors.^19,23^ These challenges are exacerbated by the opacity in deep models, where the large parameter space and complex paths for information flow make it difficult to pinpoint which features contribute to predictions. Furthermore, models are susceptible to external noise, including physiological fluctuations and imaging artefacts, which can obscure true signal patterns. This complexity underscores the need for future research focused on investigating the impact of confounders, simulating noise effects and improving the explainability of DL models.

### Challenges in pathological brain age estimation

Consistent with Cole *et al*.’s findings, we also observed a disparity in BAG (4.3-year difference) between HCs and pwMS.^13^ In MS, and at higher EDSS scores (> 4.0), the strength of correlation between EDSS and estimated brain age or BAG became weak and insignificant, more evidently for DL-SFCN, suggesting a possible residual effect of the training data being derived from healthy brains. It is also plausible that the typical patterns of brain ageing no longer apply in the same way, and that the variability in brain age estimation increases at a higher EDSS, rendering it more useful in lower disability groups. Therefore, it seems crucial to emphasize the preconceived algorithmic representation of age-dependent neurodegeneration in any brain age model, especially when the input data is pathological in nature. We also observed a difference between lesion-filled and non-lesion-filled data in the DL-derived brain age estimates, which raises concerns about a potential volumetric bias that might be affecting the results. It suggests that the presence of lesions in the T_1_w images, or the lesion filling processing, may have an impact on brain age estimates, leading to variations in the predicted age. Furthermore, we only included brain MRIs, and not spinal cord, in our analyses. Additionally, our findings also suggested that FreeSurfer version may influence variance, but does not significantly affect brain age prediction accuracy. This variability underscores the importance of harmonized preprocessing pipelines, particularly in multi-site studies and in small datasets.

### Association of brain age with clinical disability and disease duration

The results of the LME analysis indicated an association between disease duration and brain age estimation when applying the ML-1118 model, whereas EDSS exhibited a stronger association when using the DL-SFCN-derived estimates. Notably, both models shared the same conclusion for the significance of predictor variables, except for PPMS and RRMS, which remained non-significant in both models. In ML-1118, pwMS receiving high-efficacy DMTs displayed a statistically significant 0.62-year reduction in estimated brain age compared with untreated individuals. This finding is intriguing, considering that the non-treatment group had a significantly lower chronological age. However, these results should be interpreted with caution since people receiving active treatment may tend to be younger and the observed reduction in brain age might be overestimated and skewed due to a possible healthy selection bias. The DL-SFCN model had a higher marginal *R*-squared value of 0.962, indicating a better overall fit of variance in brain age. Ultimately, both models offered insights into factors influencing brain age with age and scanner type appearing as significant predictors in both models, while other variables such as scanner manufacturer had varying degrees of impact. The high ICC values suggest that site-specific factors play a significant role in the variability, meaning that brain age estimates are more consistent within a specific site, but vary across sites. Site-specific factors such as different MRI scanners, imaging protocols or data acquisition environments, may play a role in these variability issues. The DL-SFCN model had a lower total variability and slightly lower site-specific variability compared with the ML-1118 model, suggesting that it may provide more stable and consistent brain age estimates across sites. The DL-SFCN model demonstrated a slightly better overall performance with regard to *R*-squared. The EDSS score demonstrated a more substantial correlation, albeit with a slightly milder impact on brain age estimates when employing DL-SFCN, suggesting potential variances in feature penetrance and processing at a specific stage within the analysis. Nevertheless, BAG-derived from DL-SFCN exhibited no significant differences in co-correlative comparison with ML-1118-derived BAG at EDSS ≤3.5. Additionally, BAG did not demonstrate superior performance in terms of association with clinical disability when compared with estimated brain age.

### Brain age prediction and disability progression in MS: insights from ML and DL

Cole *et al*. linked BAG, DNA methylation-based age and leucocyte telomere length in HCs to mortality.^22^ Recently, Brier *et al*. reported that brain age and patient-reported disease steps were predictive of disability progression over time, a conclusion consistent with Cole *et al*.'s earlier findings, which highlighted a link between an older-appearing brain at baseline and accelerated disability progression in MS.^9,12^ Our findings align with this, but as Manouchehrinia *et al*. also noted, the EDSS scores primarily depended on disease duration and age, lacking precision at times.^37^ Numerous previous brain age studies using traditional ML approaches have identified limitations related to input data, longitudinal consistency, generalizability and the interplay between feature selection and algorithm choice, yet few have comprehensively examined the variations in feature extraction methods between traditional ML- and DL-based models for brain age in MS.^20,38-40^ However, these methods typically require less data and are often less computationally expensive. In contrast, DL models leverage automatic feature selection, requiring access to a very large amount of data. They provide a highly versatile tool for image analysis, especially when combined with ensemble approaches, which may enhance performance by integrating predictions from multiple models, but also facilitates a more comprehensive approach to explainability.^41-43^

### Scanner-induced variability and model robustness

Our study identified limitations tied to scanner variability and potential biases from noise, field strength or manufacturer, not fully explored in prior research. Stankiewicz *et al*. noted differences at 1.5 T and 3 T, with 3 T potentially enhancing sensitivity to lesion assessment.^44^ DL auto-extraction isolates predictive features, but may amplify bias or noise related to class and group differences during early training, ultimately resulting in classification errors.^45^ Hence, additional research is crucial to fully grasp how scanner variability, field strength and scanner quality affects the estimation of brain age, especially in longitudinal data.^46^ Inter-scanner heterogeneity at the group and individual levels still pose a large confounding effect for advanced imaging research, making clinical implementation of brain age and multi-site studies particularly challenging. Inter- and intra-scanner variability can be mitigated by accounting for semi-controllable confounders such as scan positioning, time of day and by implementing standardized procedures.^47^ Valdes-Hernandez *et al*. have recently demonstrated that utilizing the link between clinical MRIs and high-resolution research-grade MP-RAGEs obtained with ‘synthSR’ allows for the prediction of brain age from clinical-grade MRIs, regardless of their modality, in-plane resolution, slice thickness or orientation.^48^ In our comparison, analyses obtained from eight different scanners resulted in several significant differences in mean brain age estimates. These findings became accentuated when all EDSS categories at all time points were included in the analysis. When EDSS was categorized into three main groups (EDSS ≤3.5, 4–6 and >6), significant inter-scanner differences in mean estimates were only detected in the ML-1118. We also found an intra-model difference in brain age estimates for ML-1118 at EDSS ≤3.5 for both 1.5 T and 3 T data.

The robustness of the DL-SFCN brain age model across different field strengths and scanners likely stems from its segmentation process and architecture. Previous studies, such as Wittens *et al.*, detected structural variability in volumetric measurements attributed to differences in magnetic field strength, with a preference for affecting smaller structures like the hippocampi.^47^ Similarly, we observed a significant positive impact on BAG at higher magnetic field strengths for ML-1118. An explanation for this may reflect Chu *et al*.’s report of a lower mean brain parenchymal volume at 3 T compared with 1.5 T in MS.^49^ This finding also highlights DL-SFCN’s ability to learn features that remain invariant across scanners, even without specific adjustments during training. Thus, ML-1118 might be influenced by field strength variations, possibly due to volumetric bias or due to the relative weighted differences in feature selection and trainable parameters. Furthermore, while field strength impacts MRI resolution and signal-to-noise ratio, the DL-SFCN model’s design may allow it to generalize better, maintaining consistent performance despite these variations. Further studies employing techniques like salient object detection or layer-wise relevancy propagation and other types of backpropagation saliency techniques may shed some light on this.

The predictive accuracy of the DL-SFCN tuned with a terminal regression classifier improved performance, as opposed to SoftMax, on data originating from unknown scanners when compared with other studies with similar size and complexity.^28^ Considering our findings, it is plausible that DL might have a slightly stronger clinical correlation when the disability is low, reflecting a higher predictive power for ‘healthier’ looking brains. Attenuating the correlative dissociation at higher EDSS scores by harmonization, transfer- or federated learning may play an important role in the future of neuroimaging and multi-site imaging studies.

## Conclusion

In conclusion, the DL-SFCN model showed comparable performance to the traditional ML model in terms of clinical associations. The utilization of DL techniques in MRI data interpretation offers promising prospects, particularly in reducing scanner-induced biases. Further investigations are essential to fully explore the clinical significance of DL-based brain age as an imaging marker with prognostic capabilities, its utility in assessing treatment response and its ability to quantify the burden of neurodegeneration.

### Limitations

Our study has several limitations that need to be addressed. Brain age estimation, including BAG, may suffer from overfitting and underfitting issues, and its value can be limited due to confounders like chronological age, scanners and field strength. Our results are estimates, not true biological measurements, and we used EDSS for comparisons through correlations and LME associations. The inter-rater reliability of the EDSS was not assessed, but its reliability is acknowledged to be less than ideal.^26^ The absence of some EDSS scores may reduce statistical power and therefore affect the integrity of clinical associations. We restricted the inclusion of EDSS scores to within 6 months of the MRI, but this also represents a confounding factor due to the nature of the disease and the possibility of relapses or clinical progression in that time. Non-correctable and naturally occurring confounders in the MRI dataset included variable time-of-day scanning and physiological factors affecting brain volume. Software upgrades and changes to the MRI acquisition in terms of sequence parameters might also have influenced our results. Notably, FreeSurfer version differences between clinical sites were identified as a potential source of variability. Our sub-analysis showed no significant difference in the model's predictive performance between versions 5.3.0 and 7.1.0; however, differences in variance suggest that preprocessing inconsistencies may affect results, particularly in smaller datasets. Additionally, the possibility of healthy selection bias must be considered, as patients who transitioned to high-efficacy DMTs after 3.61 years of follow-up may represent a healthier subset of the population. This bias could underestimate the progression risk or clinical severity observed in the broader MS population. No correction for hydration status or scan synchronization by time of day was performed, and naturally, different operators were used during routine positioning. Degradation of the magnet coil over time may have affected the image quality.^50^

## Supplementary Material

fcaf152_Supplementary_Data

## Data Availability

The pretrained weights and model setup for the DL-SFCN are available for download at GitHub (https://github.com/estenhl/pyment-public.git). The ML-1118 can also be downloaded (https://github.com/tobias-kaufmann/brainage). Furthermore, an automated MRI processing pipeline with a quality control and dataset generator was also developed to assist in data handling and is available for download at GitHub (https://github.com/Laroz220/BSL). The study's supporting data can be accessed upon reasonable request by a qualified researcher, pending approval of a proposal by the corresponding author. This includes de-identified individual participant data, a data dictionary and other relevant scripts and documents. The MRI data is regarded as sensitive material and will not be made publicly available.
