# Supplementary material for: Brain age in multiple sclerosis: a study with deep learning and traditional machine learning
Source: Brain Commun. 2025 Apr 18;7(3):fcaf152. doi: 10.1093/braincomms/fcaf152 (PMC12056726; doi:10.1093/braincomms/fcaf152)
Supplement: fcaf152_Supplementary_Data [file fcaf152_supplementary_data.pdf]

Supplementary Table 1 MRI Scanner Specifications and Acquisition Parameters

**MRI acquisition parameters**								
**Scanner/ parameter:**	**GE 750**	**GE Premier**	**Siemens Aera**	**Siemens Avanto /Karolinska**	**Siemens Avanto /Oslo**	**Siemens Skyra**	**Siemens Trio**	**Siemens Trio**
**Sequence**	Sagittal 3D fast SPGR (BRAVO)	Sagittal 3D GRE (MPRAGE)	Axial 3D GRE (MPRAGE)	Axial 3D GRE (MPRAGE)	3D Sagittal GRE (MPRAGE)	Sagittal 2D GRE (MPRAGE)	Axial 3D GRE (MPRAGE)	Axial 3D GRE (MPRAGE)
**Field strength, T**	3·0	3·0	1·5	1·5	1·5	3·0	3·0	1·5
**Voxel size, mm**	Isotropic 1·0	Isotropic 0·8	1·0x1·0x1·5	1·0x1·0x1·5	1·25x1·25x1·2	0·5x0·5x1·0	1·0x1·0x1·5	1·0x1·0x1·5
**Field -of-view, mm**	256x256	240x240	226x250	249x249	192x192	240x256	249x249	-
**Echo time, ms**	3·18	3·01	3·02	3·55	3·61	2·98	3·39	7·0
**Repetition time, ms**	8·16	2356	1900	1900	2400	2300	1900	13·5
**Inversion time, ms**	450	950	1100	1100	1000	850	900	300
**Flip angle, Degrees**	12	8	15	15	8	8	9	15
**Slices, No.**	188	240	160	160	160	176	160	-

BRAVO: Brain volume imaging; **GRE**: Gradient echo; **MPRAGE**: Magnetization-prepared rapid gradient-echo; **T**: Tesla; **mm**: millimeter; **ms**: milliseconds.

MRI acquisition parameters used in the MS-cohort, including information on the scanner type, field strength (T), voxel size, field-of-view (FOV), echo time (TE), repetition time (TR), inversion time (TI), flip angle, and the number of slices acquired. The scanners used in the study include Siemens Vision, Aera, Avanto, Trio, and GE Premier, with field strengths ranging from 1.5T to 3.0T. The voxel sizes are consistent across the scanners, except for GE Premier, and the FOV varies among them. The TE and TR parameters differ slightly between the scanners, as do the inversion times and flip angles.

Supplementary Table 2 Architectural Overview of Regression3DSFCN: A 3D Neural Network for Regression Tasks

1. Regression3DSFCN/inputs (InputLayer): This layer represents the input data with a shape of (None, 167, 212, 160), where None indicates variable batch size.
2. Regression3DSFCN/expand_dims (Reshape): Reshapes the input to (None, 167, 212, 160, 1) by adding an extra dimension.
3. Regression3DSFCN/block1/conv (Conv3D): The first convolutional layer with 32 filters, resulting in an output shape of (None, 167, 212, 160, 32). It has 896 trainable parameters.
4. Regression3DSFCN/block1/norm (BatchNormalization): Batch normalization layer after the first convolutional layer.
5. Regression3DSFCN/block1/relu (Activation): Activation function (Rectified Linear Unit) applied after batch normalization.
6. Regression3DSFCN/block1/pool (MaxPooling3D): Max-pooling layer reducing the spatial dimensions to (None, 83, 106, 80, 32).
7. Regression3DSFCN/block2/conv (Conv3D): The second convolutional layer with 64 filters, resulting in an output shape of (None, 83, 106, 80, 64). It has 55,360 trainable parameters.
8. Regression3DSFCN/block2/norm (BatchNormalization): Batch normalization layer after the second convolutional layer.
9. Regression3DSFCN/block2/relu (Activation): Activation function applied after batch normalization.
10. Regression3DSFCN/block2/pool (MaxPooling3D): Max-pooling layer reducing the spatial dimensions to (None, 41, 53, 40, 64).
11. Regression3DSFCN/block3/conv (Conv3D): The third convolutional layer with 128 filters, resulting in an output shape of (None, 41, 53, 40, 128). It has 221,312 trainable parameters.
12. Regression3DSFCN/block3/norm (BatchNormalization): Batch normalization layer after the third convolutional layer.
13. Regression3DSFCN/block3/relu (Activation): Activation function applied after batch normalization.
14. Regression3DSFCN/block3/pool (MaxPooling3D): Max-pooling layer reducing the spatial dimensions to (None, 20, 26, 20, 128).
15. Regression3DSFCN/block4/conv (Conv3D): The fourth convolutional layer with 256 filters, resulting in an output shape of (None, 20, 26, 20, 256). It has 884,992 trainable parameters.
16. Regression3DSFCN/block4/norm (BatchNormalization): Batch normalization layer after the fourth convolutional layer.
17. Regression3DSFCN/block4/relu (Activation): Activation function applied after batch normalization.
18. Regression3DSFCN/block4/pool (MaxPooling3D): Max-pooling layer reducing the spatial dimensions to (None, 10, 13, 10, 256).
19. Regression3DSFCN/block5/conv (Conv3D): The fifth convolutional layer with 256 filters, resulting in an output shape of (None, 10, 13, 10, 256). It has 1,769,728 trainable parameters.
20. Regression3DSFCN/block5/norm (BatchNormalization): Batch normalization layer after the fifth convolutional layer.
21. Regression3DSFCN/block5/relu (Activation): Activation function applied after batch normalization.
22. Regression3DSFCN/block5/pool (MaxPooling3D): Max-pooling layer reducing the spatial dimensions to (None, 5, 6, 5, 256).
23. Regression3DSFCN/top/conv (Conv3D): A convolutional layer with 64 filters, resulting in an output shape of (None, 5, 6, 5, 64). It has 16,448 trainable parameters.
24. Regression3DSFCN/top/norm (BatchNormalization): Batch normalization layer after the top convolutional layer.
25. Regression3DSFCN/top/relu (Activation): Activation function applied after batch normalization.
26. Regression3DSFCN/top/pool (GlobalAveragePooling3D): Global average pooling layer reducing the spatial dimensions to (None, 64).
27. Regression3DSFCN/top/dropout (Dropout): Dropout layer for regularization.
28. Regression3DSFCN/predictions (Dense): The final dense layer with one output unit for regression, having 65 trainable parameters.
29. Regression3DSFCN/restrict/relu (ReLU): Rectified Linear Unit applied after predictions.
30. tf.math.add (TFOpLambda): A lambda layer for adding tensors.

The "Regression 3D SFCN" neural network is specifically designed for processing 3D data and is tailored for regression tasks. The initial "InputLayer" labeled as "Regression3DSFCN/inputs" receives the 3D input data, which has a shape of (None, 167, 212, 160). The "None" dimension indicates that the network can handle variable batch sizes. To prepare the data for further processing, the "Reshape" layer labeled as "Regression3DSFCN/expand_dims" adds an extra dimension to the data, resulting in a shape of (167, 212, 160, 1). The network proceeds through a series of convolutional layers, batch normalization layers, activation functions (ReLU), and max- pooling layers. Each "block" in the architecture, denoted as "Regression3DSFCN/blockX," consists of these components, with different numbers of filters and specific shapes for each block. The "top" section of the network, labeled as "Regression3DSFCN/top," comprises additional convolutional, batch normalization, and ReLU layers. The global average pooling layer "GlobalAveragePooling3D" is used to further reduce the spatial dimensions, resulting in a shape of (None, 64). This layer aggregates information from the entire input volume into a fixed-size representation. To prevent overfitting, a "Dropout" layer is applied, which randomly deactivates some neurons during training. The final layer, "Regression3DSFCN/predictions," is a dense layer with one output unit. This layer is responsible for generating regression predictions. After predictions are made, a ReLU activation function ("Regression3DSFCN/restrict/relu") is applied for potential further processing. Finally, a lambda layer ("tf.math.add (TFOpLambda)") is used for adding tensors.

Supplementary Figure 1 Reliability through Robust LME Modeling and Outlier Mitigation

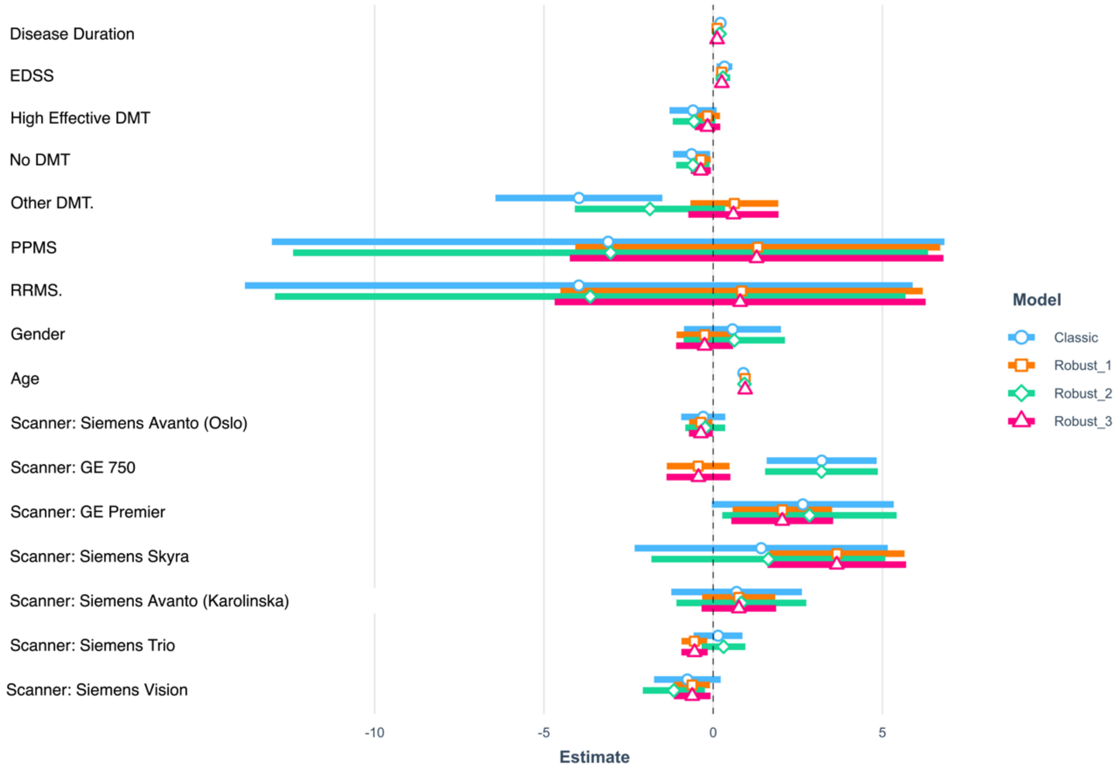

EDSS: Expanded disability status scale; **DMT**: Disease-modifying treatment; **PPMS**: Primary progressive multiple sclerosis; **RRMS**: Relapsing-remitting multiple sclerosis

An exploratory comparison of non-robust and robust linear mixed-effects (LME) models to optimize model fitness. The DL-SFCN-derived brain age was used as the response variable. In the robust approach using the ´rlmer´ function in R, each iteration varied in tuning parameters for the Huber psi-function, which controls the influence of outliers. Each fixed effect (y-axis) is represented by four non-scaled estimates on the x-axis for model comparison. In the non-robust LME model (n = 3682), disease duration ($\beta = 0.45$, $p < 0.001$), EDSS ($\beta = 0.22$, $p = 0.002$), and age ($\beta = 0.35$, $p < 0.001$) were significant predictors of brain age. In the robust models (Robust 1, Robust 2, Robust 3; n = 3682), the effect sizes for these predictors remained stable for disease duration ($\beta = 0.50$), EDSS ($\beta = 0.20$), and age ($\beta = 0.30$).

As the ´rlmer´ function in R does not provide p-values, statistical significance was inferred from the stability and magnitude of the effect sizes across the robust models.[1] Specifically, statistical significance was assessed based on the consistency of the estimated coefficients (β) and their stability across different robust model specifications. In robust modeling, the absence of p-values is compensated by the robustness of the parameter estimates, which are less influenced by outliers and more reflective of the underlying relationships in the data. The consistency of effect sizes provides strong evidence for the reliability of the predictors (disease duration, EDSS, and age) in explaining brain age, thus supporting the robustness of the findings without relying on traditional p-value-based significance testing.

However, the robust methods focus more on reducing the influence of outliers and do not follow the same statistical assumptions as classical models. This is why rlmer doesn't give p-values in the standard sense — it doesn't rely on traditional statistical methods like normality or fixed thresholds for significance. It’s based on more robust statistics (like Huber’s psi function), which provide stable coefficient estimates even with outliers, but they don't lend themselves easily to p-value calculation in the traditional way.[2]

Results indicate that robust modeling did not substantially alter brain age estimates. A similar pattern was observed when using ML-1118-derived brain age.

Supplementary Table 3 Co-Correlation Analysis of DL/ML-Derived Brain Age Gap and EDSS: Consistent Null Hypothesis Retention

Method	Method	Df	P-value	Result
Pearson and Filon's z (1898)	z = -0·6885	N/A	0·4912	Null hypothesis retained
Hotelling's t (1940)	t = -0·6883	3685	0·4913	Null hypothesis retained
Williams' t (1959)	t = -0·6882	3685	0·4913	Null hypothesis retained
Olkin's z (1967)	z = -0·6885	N/A	0·4913	Null hypothesis retained
Dunn and Clark's z (1969)	z = -0·6882	N/A	0·4912	Null hypothesis retained
Hendrickson, Stanley, and Hills' (1970) modification of Williams' t (1959)	t = -0·6883	3685	0·4913	Null hypothesis retained
Steiger's (1980) modification of Dunn and Clark's z (1969) using average correlations	z = -0·6882	N/A	0·4913	Null hypothesis retained
Meng, Rosenthal, and Rubin's z (1992)	z = -0·6882	N/A	0·4913	Null hypothesis retained
Hittner, May, and Silver's (2003) modification of Dunn and Clark's z (1969) using a backtransformed average Fisher's (1921) Z procedure	z = -0·6882	N/A	0·4913	Null hypothesis retained
Zou's (2007) confidence interval	95% confidence interval for r.jk - r.jh: -0·0385 0·0185	N/A	N/A	Null hypothesis retained (Interval includes 0)

A co-correlation plot between DL-/ML-derived BAG and EDSS. The table includes the test name, the test statistic (t/z), degrees of freedom (df), p-value, the result of the test (whether the null hypothesis is retained or not), and the 95% confidence interval for the difference r.jk - r.jh (if applicable).[3] In all cases, the null hypothesis is retained, indicating that there is no significant difference between r.jk and r.jh. The confidence intervals for the difference also include zero, further supporting the null hypothesis. The following results were similar for brain age.

Supplementary Figure 2 Correlations Between Chronological Age and Brain Age: Unadjusted

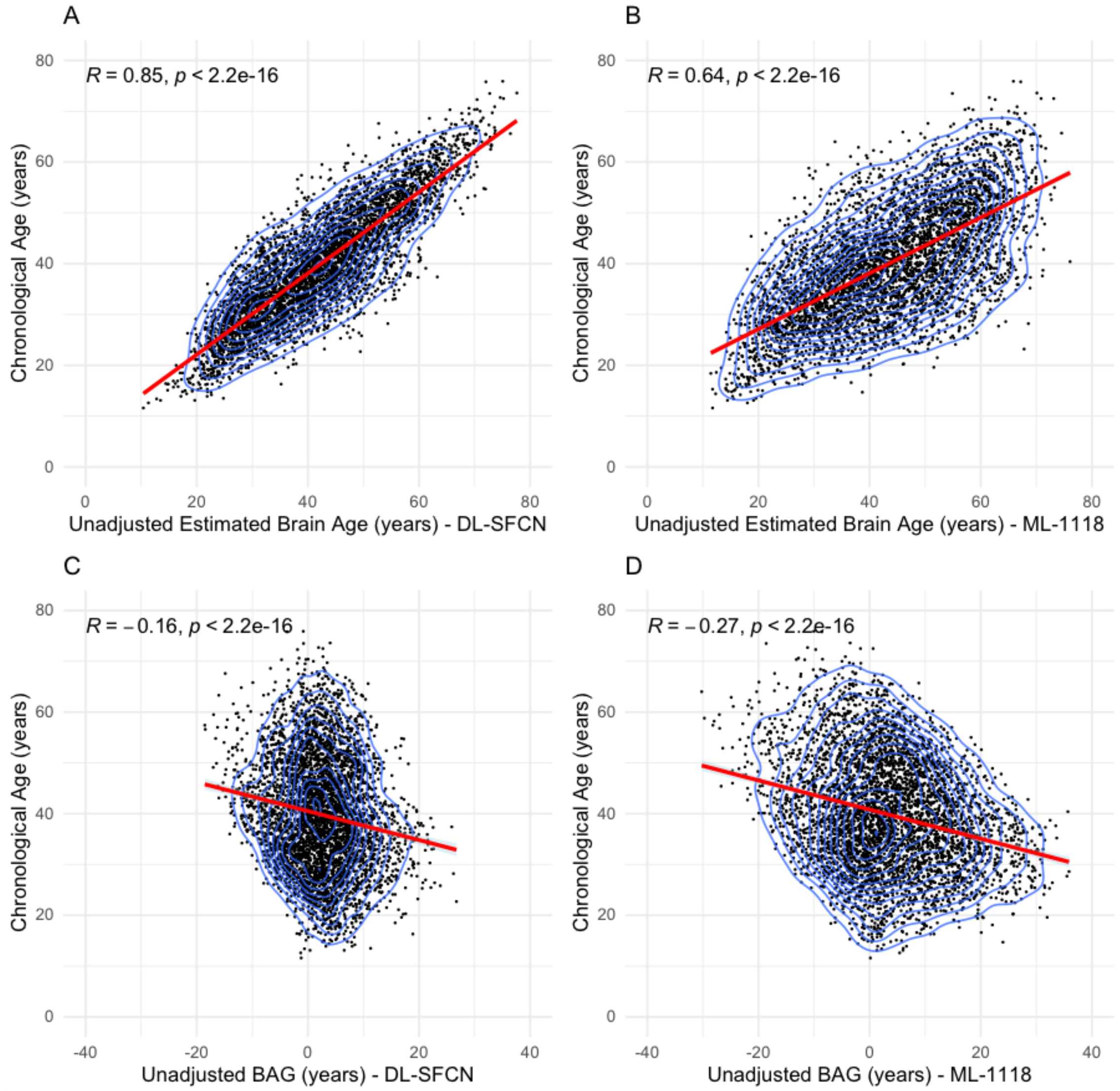

EDSS: Expanded disability status scale; **DL-SFCN**: Deep-learning simple fully convolutional network; **ML-1118:** Machine learning 1118 features; **BAG**: Brain age gap; **R**: Pearson's correlation coefficient.

Scatter plot with a layered density outline (A-D) depicting the relationship between chronological age and estimated brain age in years using two models, DL-SFCN (Panel A; R = 0.85, p < 0.001, n = 4584) and ML-1118 (Panel B; R = 0.64, p < 0.001, n = 4584), using longitudinal data. Section C-D represent the correlation between DL-SFCN- (Panel C; R = -0.16, p < 0.001) and ML-1118- derived (Panel D; R = -0.27, p < 0.001) BAG to chronological age, respectively. The brain age estimations are unadjusted for age, age^2, scanner, and gender.

Supplementary Table 4 Chronological Age Group Analysis of Brain Age Variability

	ML-1118	DL-SFCN			
**Chronological Age Group / Brain Age**	Variability	Variability	F	95% CI	**P-value**
**All Age Groups (chrono-age)**	219·38	153·01	1·43	1·35-1·52	**< 0·0001**
**Age 10-20**	53·59	28·05	1·91	1·33-2·75	**0·00056**
**Age 21-30**	102·38	28·49	3·59	3·12-4·14	**< 0·0001**
**Age 31-40**	119·34	41·66	2·87	2·58-3·18	**< 0·0001**
**Age 41-50**	104·36	39·04	2·67	2·37-3·00	**< 0·0001**
**Age 51-60**	84·74	35·67	2·38	2·03-2·79	**< 0·0001**
**Age 61-70**	58·05	28·71	2·02	1·46-2·80	**< 0·0001**
**Age 71-80**	30·08	6·49	4·64	0·81-23·73	0·081
**Chronological Age Group / BAG**					
**All Age Groups (chrono-age)**	95·59	29·76	3·21	3·03-3·40	**< 0·0001**
**Age 10-20**	43·10	19·33	2·28	1·58-3·28	**< 0·0001**
**Age 21-30**	98·93	24·27	4·08	3·54-4·69	**< 0·0001**
**Age 31-40**	111·12	33·92	3·26	2·95-3·54	**< 0·0001**
**Age 41-50**	94·03	30·83	3·05	2·71-3·43	**< 0·0001**
**Age 51-60**	78·58	29·23	2·69	2·29-3·15	**< 0·0001**
**Age 61-70**	48·98	28·71	1·97	1·42-2·73	**< 0·0001**
**Age 71-80**	31·64	4·12	7·68	1·35-39·29	**0·023**

DL-SFCN: Deep-learning simple fully convolutional network; **ML-1118:** Machine learning 1118 features; **BAG**: Brain age gap; **F**: F-statistic; **CI**: Confidence interval

This table presents the variability in brain age estimates and BAG across chronological age groups for ML-1118 and DL-SFCN. Variability is quantified by the F-statistic, 95% confidence intervals, and p-values, illustrating the differences in model performance and the precision of brain age predictions. The results show significantly lower variability in DL-SFCN compared to ML-1118 across most age groups, indicating more stable predictions.

Supplementary Figure 3 Statistical Significance of Brain Age Discrepancies: Lesion-Filled vs. Raw Data

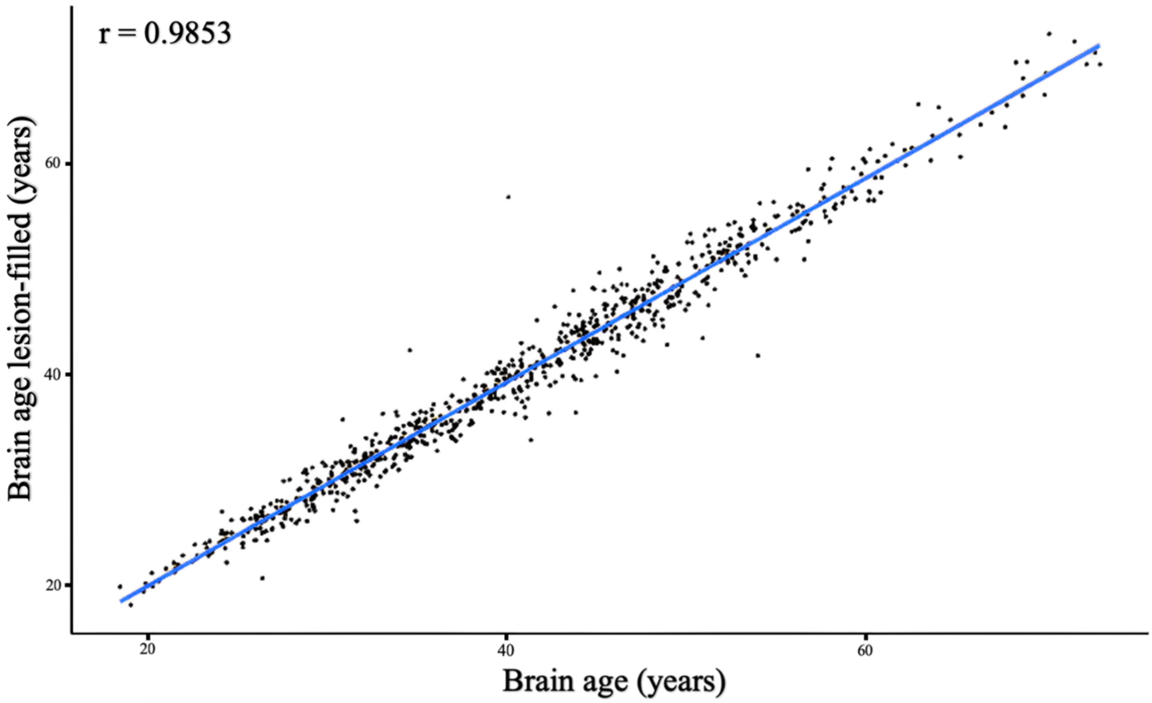

Each data point (n = 734) represents the deep learning-derived brain age estimate of an individual, calculated from both lesion-filled (y-axis) and raw T1w images (x-axis). Pearson's correlation was very high ($r = 0{\cdot}99$, $p < 0{\cdot}0001$). The test-retest reliability index, as indicated by the Intraclass Correlation Coefficient (ICC), was excellent with a value of 0·98, using a one-way random-effects model (ICC(1,1), Koo & Li (2016) classification), suggesting strong consistency in the brain age estimates across repeated measurements.[4] A t-test comparing the two groups indicated a high level of statistical significance ($t(733) = 11{\cdot}95$, $p < 0{\cdot}0001$). These findings underscore the importance of carefully considering lesion-filled data and its potential implications while interpreting brain age.

Further investigation and validations are necessary to understand the underlying mechanisms causing this discrepancy and to develop strategies for minimizing volumetric bias in brain age estimation.

Supplementary Table 5 Comparative Variability Assessment of Brain Age and BAG Predictions by ML-1118 and DL-SFCN Across Different EDSS Subgroups

	**ML-1118**	**DL-SFCN**			
**EDSS category / Brain age**	Variability	Variability	F	95% CI	p-value
**EDSS (all)**	219·38	153·01	1·43	1·35-1·52	**< 0·0001**
**EDSS 3.5 or less**	200·02	135·84	1·47	1·37-1·58	**< 0·0001**
**EDSS 4.0 - 6.0**	189·22	141·94	1·33	1·10-1·61	**0·0031**
**EDSS above 6.0**	159·03	127·13	1·25	0·89-1·75	0·19
**EDSS category / BAG**					
**EDSS (all)**	95·59	29·76	3·21	3·03-3·40	**< 0·0001**
**EDSS 3.5 or less**	91·72	27·88	3·29	3·07-3·53	**< 0·0001**
**EDSS 4.0 - 6.0**	88·78	30·53	2·91	2·40-3·52	**< 0·0001**
**EDSS above 6.0**	84·93	31·30	2·71	1·93-3·80	**< 0·0001**

EDSS: Expanded disability status scale; **IQR**: Interquartile range; **DL-SFCN**: Deep-learning simple fully convolutional network; **ML-1118**: Machine learning 1118 features; **BAG**: Brain age gap; **F**: F-statistic

A variability comparison analysis of brain age and chronological age in different EDSS groups. The table presents the significance of variability differences between ML-1118 and DL-SFCN using an F-test for variance comparison. The F-statistic, 95% CI, and p-values are reported.

Supplementary Table 6 Predictors and Variability in Uncorrected Brain Age: DL-SFCN

Brain Age (DL-SFCN uncorrected)

**Predictors**	**Estimates**	**CI**	**Statistic**	**P-value**
(Intercept)	9·56	5·20 – 13·93	4·29	**< 0·0001**
Disease Duration	0·12	0·08 – 0·15	5·90	**< 0·0001**
EDSS	0·28	0·18 – 0·38	5·60	**< 0·0001**
DMT Class [Highly effective]	-0·25	-0·54-0·04	-1·66	0·097
DMT Class [No treatment]	-0·40	-0·63 – -0·17	-3·37	**0·00076**
DMT Class [Other]	0·12	-0·91 – 1·15	0·23	0·82
Phenotype [PMS]	0·98	-3·27 – 5·23	0·45	0·65
Phenotype [RRMS]	0·26	-3·96 – 4·48	0·12	0·90
Gender	1·35	0·72 – 1·99	4·18	**< 0·0001**
Age	0·83	0·80 – 0·86	56·18	**< 0·0001**
Scanner [Avanto]	-0·30	-0·57 – -0·03	-2·14	**0·032**
Scanner [GE 750]	-3·03	-3·74 – -2·31	-8·30	**< 0·0001**
Scanner [GE Premier]	-1·90	-3·06 – -0·75	-3·23	**0·0012**
Scanner [Siemens Skyra]	-1·61	-3·19 – -0·03	-1·99	**0·046**
Scanner [Siemens Avanto]	0·77	-0·07 – 1·61	1·79	0·073
Scanner [Trio]	-5·93	-6·23 – -5·63	-38·53	**< 0·0001**
Scanner [Vision]	-7·44	-7·86 – -7·03	-34·89	**< 0·0001**
**Random Effects**				
σ^2	4·78			
$\tau 00_{\text{ID\_Simple}}$	24·69			
$\tau 00_{\text{Site}}$	0·00			
N_{Site}	2			
$N_{\text{ID\_Simple}}$	1299			
**Observations**	3682			
**Marginal R^2 / Conditional R^2**	0·957 / NA			

CI: confidence interval; **Estimate**: The estimated coefficients for each predictor, β1, a fixed-effect measure; **EDSS**: Expanded disability status scale; **DMT**: Disease-modifying treatment; **DL-SFCN**: Deep-learning simple fully convolutional network; **PMS**: Primary progressive multiple sclerosis; **RRMS**: Relapsing-remitting multiple sclerosis; **Marginal R2**: Provides the variance explained only by fixed effects.

An overview of the predictive factors and their corresponding effect measure (estimate), confidence intervals, statistics (t-value), and p-values for brain age in MS using uncorrected DL-SFCN-derived estimates.

Supplementary Table 7 Predictors and Variability in Uncorrected Brain Age Gap: DL-SFCN

Brain Age Gap (DL-SFCN uncorrected)

Predictors	Estimates	CI	Statistic	P-value
(Intercept)	9·56	5·20 – 13·93	4·29	**< 0·0001**
Disease Duration	0·12	0·08 – 0·15	5·90	**< 0·0001**
EDSS	0·28	0·18 – 0·38	5·60	**< 0·0001**
DMT Class [Highly effective]	-0·25	-0·54-0·04	-1·66	0·097
DMT Class [No treatment]	-0·40	-0·63 – -0·17	-3·37	**0·00076**
DMT Class [Other]	0·12	-0·91 – 1·15	0·23	0·82
Phenotype [PMS]	0·98	-3·27 – 5·23	0·45	0·65
Phenotype [RRMS]	0·26	-3·96 – 4·48	0·12	0·90
Gender	1·35	0·72 – 1·99	4·18	**< 0·0001**
Age	-0·17	-0·20 – -0·14	-11·37	**< 0·0001**
Scanner [Avanto]	-0·30	-0·57 – -0·03	-2·14	**0·032**
Scanner [GE 750]	-3·03	-3·74 – -2·31	-8·30	**< 0·0001**
Scanner [GE Premier]	-1·90	-3·06 – -0·75	-3·23	**0·0012**
Scanner [Siemens Skyra]	-1·61	-3·19 – -0·03	-1·99	**0·046**
Scanner [Siemens Avanto]	0·77	-0·07 – 1·61	1·79	0·073
Scanner [Trio]	-5·93	-6·23 – -5·63	-38·53	**< 0·0001**
Scanner [Vision]	-7·44	-7·86 – -7·03	-34·89	**< 0·0001**
**Random Effects**				
σ^2	4·78			
$\tau 00_{ID\_Simple}$	24·69			
$\tau 00_{Site}$	0·00			
N_{Site}	2			
N_{ID_Simple}	1299			
**Observations**	3682			
**Marginal R^2 / Conditional R^2**	0·957 / NA			

CI: confidence interval; **Estimate**: The estimated coefficients for each predictor, β1, a fixed-effect measure; **EDSS**: Expanded disability status scale; **DMT**: Disease-modifying treatment; **DL-SFCN**: Deep-learning simple fully convolutional network; **PMS**: Primary progressive multiple sclerosis; **RRMS**: Relapsing-remitting multiple sclerosis; **Marginal R2**: Provides the variance explained only by fixed effects.

An overview of the predictive factors and their corresponding effect measure (estimate), confidence intervals, statistics (t-value), and p-values for brain age in MS using uncorrected DL-SFCN-derived estimates.

Supplementary Table 8 Predictors and Variability in Uncorrected Brain Age: ML-1118

Brain Age (ML-1118 uncorrected)

Predictors	Estimates	CI	Statistic	P-value
(Intercept)	17·58	9·77 – 25·39	4·42	**< 0·0001**
Disease Duration	0·21	0·15 – 0·28	6·31	**< 0·0001**
EDSS	0·30	0·12 – 0·48	3·33	**0·00089**
DMT Class [Highly effective]	-0·47	-1·01 – 0·06	1·74	0·081
DMT Class [No treatment]	-0·71	-1·13 – -0·29	-3·31	**0·00093**
DMT Class [Other]	-3·96	-5·85 – -2·07	-4·11	**< 0·0001**
Phenotype [PMS]	-3·17	-10·78 – 4·44	-0·82	0·41
Phenotype [RRMS]	-3·81	-11·37 – 3·75	-0·99	0·32
Gender	-0·24	-1·34 – 0·86	-0·43	0·67
Age	0·65	0·60 – 0·70	25·40	**< 0·0001**
Scanner [Avanto]	1·95	1·45 – 2·45	7·66	**< 0·0001**
Scanner [GE 750]	2·78	1·54 – 4·03	4·39	**< 0·0001**
Scanner [GE Premier]	3·96	1·90 – 6·01	3·77	**0·00017**
Scanner [Siemens Skyra]	3·56	0·69 – 6·43	2·44	**0·015**
Scanner [Siemens Avanto]	4·82	3·34 – 6·30	6·40	**< 0·0001**
Scanner [Trio]	6·14	5·59 – 6·69	21·81	**< 0·0001**
Scanner [Vision]	-1·01	-1·77 – -0·26	-2·64	**0·0084**
**Random Effects**				
σ^2	16·09			
τ_{00} ID_Simple	72·45			
τ_{00} Site	0·00			
N Site	2			
N ID_Simple	1299			
**Observations**	3688			
**Marginal R^2 / Conditional R^2**	0·817 / NA			

CI: confidence interval; **Estimate**: The estimated coefficients for each predictor, β1, a fixed-effect measure; **EDSS**: Expanded disability status scale; **DMT**: Disease-modifying treatment; **ML-1118**: Machine learning 1118 features; **PMS**: Primary progressive multiple sclerosis; **RRMS**: Relapsing-remitting multiple sclerosis; **Marginal R2**: Provides the variance explained only by fixed effects.

An overview of the predictive factors and their corresponding effect measure (estimate), confidence intervals, statistics (t-value), and p-values for brain age in MS using uncorrected ML-1118-derived estimates.

Supplementary Table 9 Predictors and Variability in Uncorrected Brain Age Gap: ML-1118

Brain Age Gap (ML-1118 uncorrected)

Predictors	Estimates	CI	Statistic	P-value
(Intercept)	17·58	9·77 – 25·39	4·42	**< 0·0001**
Disease Duration	0·21	0·15 – 0·28	6·31	**< 0·0001**
EDSS	0·30	0·12 – 0·48	3·33	**0·00089**
DMT Class [Highly effective]	-0·47	-1·01 – 0·06	1·74	0·081
DMT Class [No treatment]	-0·71	-1·13 – -0·29	-3·31	**0·00093**
DMT Class [Other]	-3·96	-5·85 – -2·07	-4·11	**< 0·0001**
Phenotype [PMS]	-3·17	-10·78 – 4·44	-0·82	0·41
Phenotype [RRMS]	-3·81	-11·37 – 3·75	-0·99	0·32
Gender	-0·24	-1·34 – 0·86	-0·43	0·67
Age	-0·35	-0·40 – -0·30	-13·69	**< 0·0001**
Scanner [Avanto]	1·95	1·45 – 2·45	7·66	**< 0·0001**
Scanner [GE 750]	2·78	1·54 – 4·03	4·39	**< 0·0001**
Scanner [GE Premier]	3·96	1·90 – 6·01	3·77	**0·00017**
Scanner [Siemens Skyra]	3·56	0·69 – 6·43	2·44	**0·015**
Scanner [Siemens Avanto]	4·82	3·34 – 6·30	6·40	**< 0·0001**
Scanner [Trio]	6·14	5·59 – 6·69	21·81	**< 0·0001**
Scanner [Vision]	-1·01	-1·77 – -0·26	-2·64	**0·0084**
**Random Effects**				
σ^2	16·09			
$\tau 00_{ID\_Simple}$	72·45			
$\tau 00_{Site}$	0·00			
N_{Site}	2			
N_{ID_Simple}	1299			
**Observations**	3688			
**Marginal R^2 / Conditional R^2**	0·151 / 0·846			

CI: confidence interval; **Estimate**: The estimated coefficients for each predictor, β1, a fixed-effect measure; **EDSS**: Expanded disability status scale; **DMT**: Disease-modifying treatment; **ML-1118**: Machine learning 1118 features; **PMS**: Primary progressive multiple sclerosis; **RRMS**: Relapsing-remitting multiple sclerosis; **Marginal R2**: Provides the variance explained only by fixed effects.

An overview of the predictive factors and their corresponding effect measure (estimate), confidence intervals, statistics (t-value), and p-values for brain age in MS using uncorrected ML-1118-derived estimates.

Supplementary Table 10 Assessing Brain Age and BAG at grouped EDSS with ML-1118 and DL-SFCN: A Correlation Perspective

Time Point (Baseline)	**ML-1118**	**DL-SFCN**
**EDSS (All)**	EDSS (Rho, sig.)	EDSS (Rho, sig.)
**Brain Age**	**0.36, p < 0·001**	**0.33, p < 0·001**
**BAG**	**0.16, p < 0·001**	**0.16, p < 0·001**
All Time Points (0-16)	**ML-1118**	**DL-SFCN**
**EDSS (All)**	EDSS (Rho, sig.)	EDSS (Rho, sig.)
**Brain Age**	**0.36, p < 0·001**	**0.38, p < 0·001**
**BAG**	**0.13, p < 0·001**	**0.12, p < 0·001**

All Time points (0-16)	**ML-1118**	**DL-SFCN**
**EDSS ≤3.5**	EDSS (Rho, sig.)	EDSS (Rho, sig.)
**Brain Age**	**0.25, p < 0·001**	**0.26, p < 0·001**
**BAG**	**0.08, p < 0.0023**	**0.06, p < 0.035**
**EDSS 4.0 - 6.0**		
**Brain Age**	**0.24, p < 0·001**	**0.24, p < 0·001**
**BAG**	-0.01, p = 0.88	0.025, p = 0.68
**EDSS >6.0**		
**Brain Age**	0.20, p = 0·021	-0.007, p = 0.94
**BAG**	**0.29, p = 0.002**	0.13, p = 0.18

EDSS: Expanded disability status scale; **DL-SFCN**: Deep-learning simple fully convolutional network; **ML-1118**: Machine learning 1118 features; **BAG**: Brain age gap; **Rho**: Spearman's rank correlation

This table presents Spearman's rank correlation coefficients (Rho) and their statistical significance for the association between brain age, brain age gap, and EDSS at baseline and across all time points (0–16). Results are stratified by EDSS categories and analyzed separately for ML-1118 and DL-SFCN.

Supplementary Table 11 Predictors and Variability in Brain Age at EDSS 3·5 or less: DL-SFCN

Brain Age at EDSS 3·5 or less (DL-SFCN - corrected for age, age^2, scanner and sex)

Predictors	Estimates	CI	Statistic	P-value
(Intercept)	1·13	-3·17 – 5·43	0·52	0·61
Disease Duration	0·11	0·07 – 0·15	5·04	**< 0·0001**
EDSS	0·18	0·04 – 0·31	2·61	**0·0090**
DMT Class [Highly effective]	-0·05	-0·36-0·27	-0·30	**0·76**
DMT Class [No treatment]	-0·33	-0·57 – -0·09	-2·70	**0·0070**
DMT Class [Other]	-0·20	-1·35 – 0·95	-0·34	0·73
Phenotype [PMS]	0·78	-3·40 – 4·96	0·37	0·71
Phenotype [RRMS]	0·34	-3·78 – 4·47	0·16	0·87
Gender	-0·21	-0·89 – 0·46	-0·63	0·53
Age	0·94	0·91 – 0·97	59·31	**< 0·0001**
Scanner [Avanto]	-0·51	-0·81 – -0·22	-3·45	**0·00057**
Scanner [GE 750]	-0·68	-1·43 – 0·07	-1·78	0·075
Scanner [GE Premier]	1·81	0·63 – 2·98	3·01	**0·0026**
Scanner [Siemens Skyra]	3·53	1·81 – 5·25	4·03	**< 0·0001**
Scanner [Siemens Avanto]	0·41	-0·46 – 1·28	0·92	0·36
Scanner [Trio]	-0·66	-0·98 – -0·34	-4·06	**< 0·0001**
Scanner [Vision]	-0·98	-1·44 – -0·53	-4·24	**< 0·0001**
**Random Effects**				
σ^2	4·44			
$\tau 00_{ID\_Simple}$	24·21			
$\tau 00_{Site}$	0·00			
N_{Site}	2			
N_{ID_Simple}	1149			
**Observations**	3122			
**Marginal R^2 / Conditional R^2**	0·786 / 0·967			

CI: confidence interval; **Estimate**: The estimated coefficients for each predictor, β1, a fixed-effect measure; **EDSS**: Expanded disability status scale; **DMT**: Disease-modifying treatment; **DL-SFCN**: Deep-learning simple fully convolutional network; **PMS**: Primary progressive multiple sclerosis; **RRMS**: Relapsing-remitting multiple sclerosis; **Marginal R2**: Provides the variance explained only by fixed effects.

An overview of the predictive factors, stratifying by EDSS-category, and their corresponding effect measure (estimate), confidence intervals, statistics (t-value), and p-values for brain age in MS using uncorrected DL-SFCN-derived estimates.

Supplementary Table 12 Predictors and Variability in Brain Age at EDSS 4·0-6·0: DL-SFCN

Brain Age at EDSS 4·0-6·0 (DL-SFCN - corrected for age, age^2, scanner and sex)

Predictors	Estimates	CI	Statistic	P-value
(Intercept)	6·56	-6·60 – 19·71	0·98	0·33
Disease Duration	0·13	0·03 – 0·22	2·71	**0·0069**
EDSS	0·22	-0·28 – 0·73	0·87	0·38
DMT Class [Highly effective]	-0·79	-1·75-0·18	-1·60	0·11
DMT Class [No treatment]	-0·40	-1·23 – 0·44	-0·93	0·35
DMT Class [Other]	2·79	-0·93 – 6·52	1·47	0·14
Phenotype [PMS]	-1·83	-14·72 – 11·06	-0·28	0·78
Phenotype [RRMS]	-2·03	-14·84 – 10·79	-0·31	0·76
Gender	-0·34	-1·91 – 1·23	-0·43	0·67
Age	0·89	0·82 – 0·96	23·98	**< 0·0001**
Scanner [Avanto]	0·10	-0·72 – 0·93	0·25	0·80
Scanner [GE 750]	0·73	-1·50 – 2·95	0·64	0·52
Scanner [GE Premier]	-3·08	-9·56 – 3·40	-0·94	0·35
Scanner [Siemens Skyra]	-2·23	-9·12 – 4·66	-0·64	0·52
Scanner [Siemens Avanto]	0·78	-2·09 – 3·66	0·54	0·59
Scanner [Trio]	-0·13	-1·10 – 0·84	-0·26	0·80
Scanner [Vision]	0·04	-1·21 – 1·30	0·07	0·94
**Random Effects**				
σ^2	4·45			
$\tau 00_{ID\_Simple}$	26·12			
$\tau 00_{Site}$	0·00			
N_{Site}	2			
N_{ID_Simple}	229			
**Observations**	424			
**Marginal R^2 / Conditional R^2**	0·961 / NA			

CI: confidence interval; **Estimate**: The estimated coefficients for each predictor, β1, a fixed-effect measure; **EDSS**: Expanded disability status scale; **DMT**: Disease-modifying treatment; **DL-SFCN**: Deep-learning simple fully convolutional network; **PMS**: Primary progressive multiple sclerosis; **RRMS**: Relapsing-remitting multiple sclerosis; **Marginal R2**: Provides the variance explained only by fixed effects.

An overview of the predictive factors, stratifying by EDSS-category, and their corresponding effect measure (estimate), confidence intervals, statistics (t-value), and p-values for brain age in MS using uncorrected DL-SFCN-derived estimates.

Supplementary Table 13 Predictors and Variability in Brain Age at EDSS above 6·0: DL-SFCN

Brain Age at EDSS above 6·0 (DL-SFCN - corrected for age, age², scanner and sex)

Predictors	Estimates	CI	Statistic	P-value
(Intercept)	8·30	-4·29 – 20·90	1·31	0·19
Disease Duration	0·01	-0·15 – 0·16	0·09	0·93
EDSS	0·04	-1·43 – 1·51	0·05	0·96
DMT Class [Highly effective]	-0·61	-2·78-1·55	-0·56	0·58
DMT Class [No treatment]	-1·15	-3·04 – 0·74	-1·20	0·23
DMT Class [Other]	-0·62	-4·91 – 3·67	-0·29	0·77
Phenotype [RRMS]	-1·52	-4·34 – 1·30	-1·07	0·29
Gender	-1·68	-4·84 – 1·48	-1·05	0·29
Age	0·92	0·77 – 1·06	12·71	**< 0·0001**
Scanner [Avanto]	0·99	-0·98 – 2·96	0·99	0·32
Scanner [GE 750]	1·78	-2·97 – 6·53	0·74	0·46
Scanner [Trio]	0·50	-1·82 – 2·81	0·42	0·67
Scanner [Vision]	2·93	0·28 – 5·58	2·19	**0·031**
**Random Effects**				
σ^2	6·55			
$\tau 00_{ID\_Simple}$	27·15			
$\tau 00_{Site}$	0·00			
N_{Site}	2			
N_{ID_Simple}	71			
**Observations**	136			
**Marginal R^2 / Conditional R^2**	0·942 / NA			

CI: confidence interval; **Estimate**: The estimated coefficients for each predictor, β1, a fixed-effect measure; **EDSS**: Expanded disability status scale; **DMT**: Disease-modifying treatment; **DL-SFCN**: Deep-learning simple fully convolutional network; **PMS**: Primary progressive multiple sclerosis; **RRMS**: Relapsing-remitting multiple sclerosis; **Marginal R2**: Provides the variance explained only by fixed effects.

An overview of the predictive factors, stratifying by EDSS-category, and their corresponding effect measure (estimate), confidence intervals, statistics (t-value), and p-values for brain age in MS using uncorrected DL-SFCN-derived estimates.

Supplementary Table 14 Predictors and Variability in Brain Age at EDSS 3·5 or less: ML-1118

Brain Age at EDSS 3·5 or less (ML-1118 - corrected for age, age^2, scanner and sex)

**Predictors**	**Estimates**	**CI**	**Statistic**	**P-value**
(Intercept)	6·07	-1·76 – 13·90	1·52	0·13
Disease Duration	0·22	0·15 – 0·29	5·86	**< 0·0001**
EDSS	0·17	-0·08 – 0·41	1·33	0·18
DMT Class [Highly effective]	-0·46	-1·05-0·13	-1·53	0·13
DMT Class [No treatment]	-0·47	-0·92 – -0·02	-2·03	**0·043**
DMT Class [Other]	-4·77	-6·94 – -2·61	-4·33	**< 0·0001**
Phenotype [PMS]	-2·92	-10·56 – 4·72	-0·75	0·45
Phenotype [RRMS]	-3·80	-11·35 – 3·74	-0·99	0·32
Gender	0·79	-0·37 – 1·95	1·34	0·18
Age	0·90	0·85 – 0·96	32·92	**< 0·0001**
Scanner [Avanto]	-0·48	-1·03 – 0·07	-1·72	0·085
Scanner [GE 750]	3·25	1·94 – 4·56	4·87	**< 0·0001**
Scanner [GE Premier]	2·80	0·67 – 4·93	2·57	**0·010**
Scanner [Siemens Skyra]	1·54	-1·65 – 4·73	0·95	0·34
Scanner [Siemens Avanto]	0·85	-0·69 – 2·38	1·08	0·28
Scanner [Trio]	0·09	-0·51 – 0·70	0·31	0·76
Scanner [Vision]	-1·07	-1·90 – -0·23	-2·50	**0·013**
**Random Effects**				
σ^2	15·87			
τ_{00} ID_Simple	70·78			
τ_{00} Site	0·00			
N Site	2			
N ID_Simple	1149			
**Observations**	3127			
**Marginal R^2 / Conditional R^2**	0·872 / NA			

CI: confidence interval; **Estimate**: The estimated coefficients for each predictor, β1, a fixed-effect measure; **EDSS**: Expanded disability status scale; **DMT**: Disease-modifying treatment; **ML-1118**: Machine learning 1118 features; **PMS**: Primary progressive multiple sclerosis; **RRMS**: Relapsing-remitting multiple sclerosis; **Marginal R2**: Provides the variance explained only by fixed effects.

An overview of the predictive factors, stratifying by EDSS-category, and their corresponding effect measure (estimate), confidence intervals, statistics (t-value), and p-values for brain age in MS using uncorrected ML-1118-derived estimates.

Supplementary Table 15 Predictors and Variability in Brain Age at EDSS 4·0 - 6·0: ML-1118

Brain Age at EDSS 4·0 – 6·0 (ML-1118 - corrected for age, age^2, scanner and sex)

Predictors	Estimates	CI	Statistic	P-value
(Intercept)	22·93	0·63 – 45·22	2·02	**0·044**
Disease Duration	0·25	0·09 – 0·40	3·15	**0·0018**
EDSS	0·53	-0·30 – 1·37	1·25	0·21
DMT Class [Highly effective]	0·79	-0·79-2·38	0·98	0·33
DMT Class [No treatment]	0·54	-0·83 – 1·92	0·78	0·44
DMT Class [Other]	0·07	-6·08 – 6·23	0·02	0·98
Phenotype [PMS]	-16·96	-38·82 – 4·90	-1·52	0·13
Phenotype [RRMS]	-16·79	-38·53 – 4·94	-1·52	0·13
Gender	0·30	-2·36 – 2·97	0·22	0·82
Age	0·81	0·69 – 0·93	12·88	**< 0·0001**
Scanner [Avanto]	0·37	-0·97 – 1·71	0·54	0·59
Scanner [GE 750]	3·55	-0·22 – 7·32	1·85	0·065
Scanner [GE Premier]	-5·18	-16·17 – 5·82	-0·93	0·36
Scanner [Siemens Skyra]	-6·74	-18·38 – 4·90	-1·14	0·26
Scanner [Siemens Avanto]	0·67	-4·13 – 5·47	0·27	0·78
Scanner [Trio]	-0·20	-1·78 – 1·39	-0·25	0·81
Scanner [Vision]	-0·66	-2·72 – 1·41	-0·63	0·53
**Random Effects**				
σ^2	11·75			
$\tau 00_{ID\_Simple}$	76·42			
$\tau 00_{Site}$	0·00			
N_{Site}	2			
N_{ID_Simple}	229			
**Observations**	424			
**Marginal R^2 / Conditional R^2**	0·897 / NA			

CI: confidence interval; **Estimate**: The estimated coefficients for each predictor, β1, a fixed-effect measure; **EDSS**: Expanded disability status scale; **DMT**: Disease-modifying treatment; **ML-1118**: Machine learning 1118 features; **PMS**: Primary progressive multiple sclerosis; **RRMS**: Relapsing-remitting multiple sclerosis; **Marginal R2**: Provides the variance explained only by fixed effects.

An overview of the predictive factors, stratifying by EDSS-category, and their corresponding effect measure (estimate), confidence intervals, statistics (t-value), and p-values for brain age in MS using uncorrected ML-1118-derived estimates.

Supplementary Table 16 Predictors and Variability in Brain Age at EDSS above 6·0: ML-1118

Brain Age at EDSS above 6.0 (ML-1118 - corrected for age, age^2, scanner and sex)

Predictors	Estimates	CI	Statistic	P-value
(Intercept)	3·66	-14·95 – 22·27	0·39	0·70
Disease Duration	0·14	-0·09 – 0·37	1·18	0·24
EDSS	1·90	-0·22 – 4·02	1·77	0·079
DMT Class [Highly effective]	-2·32	-5·34 – 0·70	-1·52	0·13
DMT Class [No treatment]	-3·46	-6·10 – -0·82	-2·59	**0·011**
DMT Class [Other]	-4·05	-9·90 – 1·79	-1·37	0·17
Phenotype [RRMS]	-1·55	-5·67 – 2·58	-0·74	0·46
Gender	-0·05	-5·02 – 4·92	-0·02	0·99
Age	0·80	0·58 – 1·02	7·09	**< 0·0001**
Scanner [Avanto]	0·37	-2·38 – 3·13	0·27	0·79
Scanner [GE 750]	5·02	-2·34 – 12·38	1·35	0·18
Scanner [Trio]	1·09	-2·06 – 4·24	0·68	0·50
Scanner [Vision]	0·41	-3·38 – 4·19	0·21	0·83
**Random Effects**				
σ^2	11·84			
$\tau 00_{ID\_Simple}$	70·84			
$\tau 00_{Site}$	0·00			
N_{Site}	2			
N_{ID_Simple}	72			
**Observations**	137			
**Marginal R^2 / Conditional R^2**	0·895 / NA			

CI: confidence interval; **Estimate**: The estimated coefficients for each predictor, β1, a fixed-effect measure; **EDSS**: Expanded disability status scale; **DMT**: Disease-modifying treatment; **ML-1118**: Machine learning 1118 features; **PMS**: Primary progressive multiple sclerosis; **RRMS**: Relapsing-remitting multiple sclerosis; **Marginal R2**: Provides the variance explained only by fixed effects.

An overview of the predictive factors, stratifying by EDSS-category, and their corresponding effect measure (estimate), confidence intervals, statistics (t-value), and p-values for brain age in MS using uncorrected ML-1118-derived estimates.

Supplementary Table 17 Variability in Brain Age and Brain Age Gap Among MRI Scanners (ML-1118 / DL-SFCN) and Levine's Test Results

**Brain Age Variability (W) ML-1118 / DL-SFCN**	**Avanto**	**Vision**	**Siemens Skyra**	**GE Premier**	**GE 750**	**Siemens Avanto**	**Trio**	**Aera**
**Aera**	1·50 (p < 0·0001)	1·58 (p < 0·0001)	1·49 (p = 0·00018)	1·47 (p = 0·00018)	1·22 (p = 0·015)	1·53 (< 0·0001)	1·36 (p < 0·0001)	**1·46 (p < 0·0001)**
**Trio**	1·44 (p < 0·0001)	1·48 (p < 0·0001)	1·30 (p = 0·013)	1·30 (p = 0·011)	1·15 (p = 0·085)	1·36 (p = 0·0010)	**1·28 (p = 0·0011)**	1·36 (p < 0·0001)
**Siemens Avanto**	1·51 (p < 0·0001)	1·65 (p < 0·0001)	1·54 (p = 0·037)	1·45 (p = 0·045)	1·04 (p = 0·73)	**1·71 (p = 0·00025)**	1·28 (p = 0·0085)	1·44 (p < 0·0001)
**GE 750**	1·38 (p < 0·0001)	1·43 (p < 0·0001)	0·96 (p = 0·73)	1·01 (p = 0·96)	**0·92 (p = 0·38)**	1·16 (p =0·17)	1·17 (p = 0·053)	1·23 (p = 0·0088)
**GE Premier**	1·50 (< 0·0001)	1·65 (p < 0·0001)	1·24 (p = 0·62)	**1·20 (p = 0·54)**	0·94 (p = 0·58)	1·69 (p = 0·0043)	1·25 (p = 0·03)	1·42 (p = 0·00041)
**Siemens Skyra**	1·51 (p < 0·0001)	1·67 (p < 0·0001)	-	1·13 (p = 0·77)	0·90 (p = 0·40)	1·91 (p = 0·0017)	1·25 (p = 0·035)	1·43 (p = 0·00042)
**Vision**	1·62 (p < 0·0001)	**1·76 (p < 0·0001)**	1·86 (p < 0·0001)	1·83 (p < 0·0001)	1·48 (p < 0·0001)	1·86 (< 0·0001)	1·54 (p < 0·0001)	1·64 (p < 0·0001)
**Avanto**	**1·49 (p < 0·0001)**	1·53 (p < 0·0001)	1·47 (p < 0·0001)	1·46 (p < 0·0001)	1·34 (p < 0·0001)	1·48 (p < 0·0001)	1·39 (p < 0·0001)	1·45 (p < 0·0001)

**BAG Variability (W) ML-1118 / DL-SFCN**	**Siemens Avanto I**	**Siemens Vision**	**Siemens Skyra**	**GE Premier**	**GE 750**	**Siemens Avanto II**	**Siemens Trio**	**Siemens Aera**
**Aera**	4·27 (p < 0·0001)	3·32 (p < 0·0001)	4·38 (p < 0·0001)	4·41 (p < 0·0001)	2·91 (p < 0·0001)	4·01 (p < 0·0001)	3·08 (p < 0·0001)	**4·18 (p < 0·0001)**
**Trio**	3·39 (p < 0·0001)	2·48 (p < 0·0001)	2·23 (p < 0·0001)	2·29 (p < 0·0001)	2·08 (p < 0·0001)	2·34 (p < 0·0001)	**2·28 (p < 0·0001)**	2·91 (p < 0·0001)
**Siemens Avanto**	4·12 (p < 0·0001)	2·79 (p < 0·0001)	3·07 (p < 0·0001)	3·30 (p < 0·0001)	2·06 (p < 0·0001)	3·**01 (p < 0·0001)**	2·44 (p < 0·0001)	3·81 (p < 0·0001)
**GE 750**	3·63 (p < 0·0001)	2·53 (p < 0·0001)	1·91 (p < 0·0001)	2·07 (p < 0·0001)	**1·81 (p < 0·0001)**	2·27 (p < 0·0001)	2·30 (p < 0·0001)	3·16 (p < 0·0001)
**GE Premier**	4·26 (p < 0·0001)	2·80 (p < 0·0001)	6·09 (p < 0·0001)	**5·44 (p < 0·0001)**	1·86 (p < 0·0001)	3·21 (p < 0·0001)	2·39 (p < 0·0001)	4·05 (p < 0·0001)
**Siemens Skyra**	4·24 (p < 0·0001)	2·74 (p < 0·0001)	-	4·99 (p < 0·0001)	1·73 (p < 0·0001)	2·95 (p < 0·0001)	2·32 (p < 0·0001)	4·00 (p < 0·0001)
**Vision**	3·92 (p < 0·0001)	**3·08 (p < 0·0001)**	3·42 (p < 0·0001)	3·46 (p < 0·0001)	2·77 (p < 0·0001)	3·35 (p < 0·0001)	2·88 (p < 0·0001)	3·66 (p < 0·0001)
**Avanto**	**4·00 (p < 0·0001)**	3·37 (p < 0·0001)	3·76 (p < 0·0001)	3·78 (p < 0·0001)	3·21 (p < 0·0001)	3·67 (p < 0·0001)	3·23 (p < 0·0001)	3·83 (p < 0·0001)

DL-SFCN: Deep-learning simple fully convolutional network; **ML-1118**: Machine learning 1118 features; **BAG**: Brain age gap; **W**: W-statistic

This table illustrates the variability statistic (W) of brain age (top table) and BAG (bottom table) by ML-1118 and DL-SFCN at different scanners. Each scanner is listed on the x- and y-axis of the table in a reverse correlated order. Siemens Avanto I and II were located in Oslo and Karolinska, respectively. The significance of the W-statistic was obtained by using Levene's test. Siemens Skyra could not be calculated due to a low number of observations (n =14). For all other scanners, the level of variance was significantly different, as illustrated by the highly significant p-value < 0.0001.

Supplementary Figure 4 DL-SFCN: MRI Scanner differences and Its Influence on Brain Age Gap at all EDSS categories

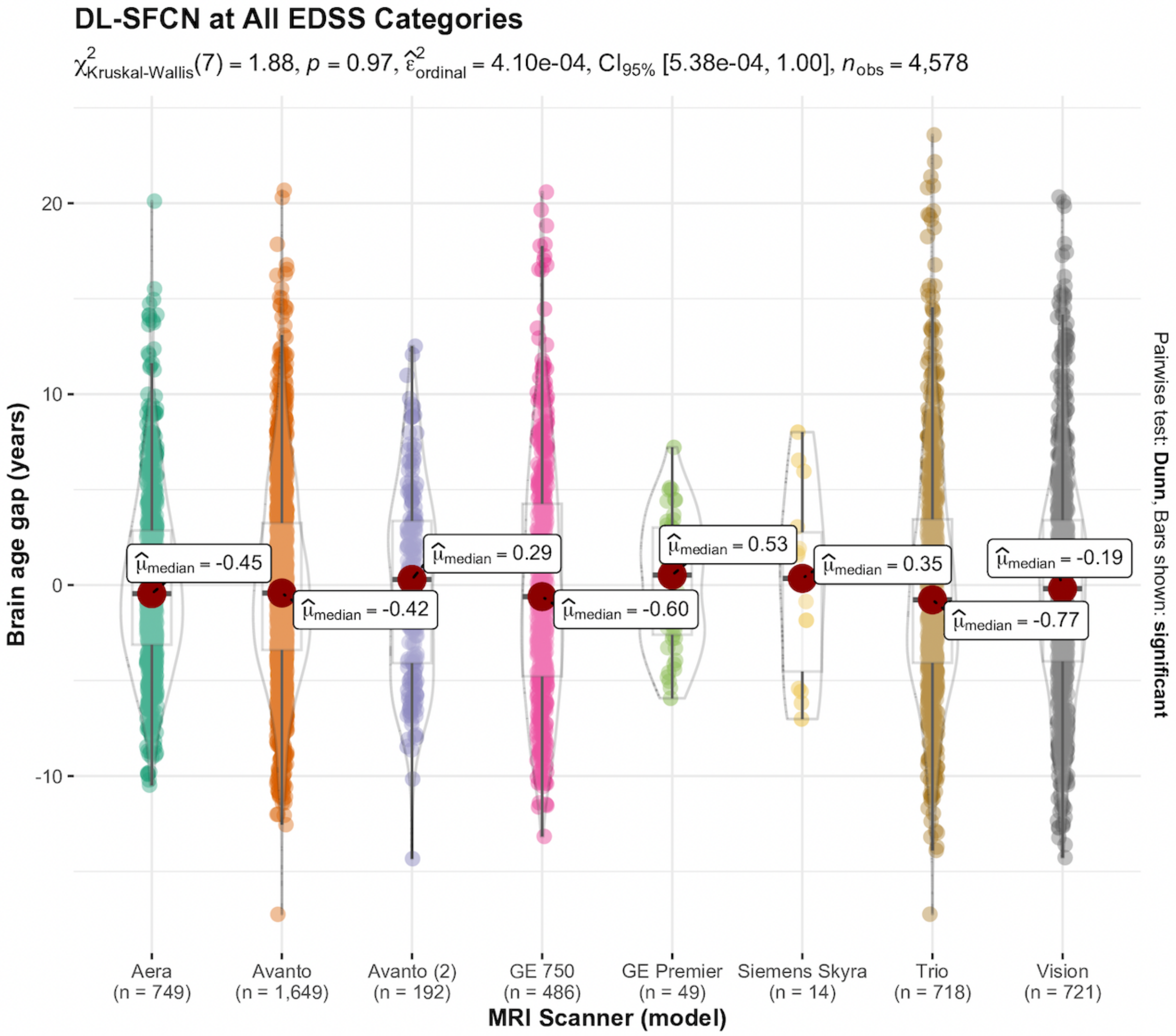

DL-SFCN: Deep-learning simple fully convolutional network; **BAG**: Brain age gap; **EDSS**: Expanded disability status scale

The Chi-Square Kruskal-Wallis Test was used for multiple comparisons of non-parametric data with unequal variances to evaluate inter-scanner differences in DL-SFCN-derived BAG across all EDSS scores (range 0–9). The test revealed statistically nonsignificant overall differences between scanner models ($X^2 = 1.88$, $p = 0.97$), with a very small effect size (ε^2ordinal <0.001, 95% CI [0.01, 1.00]). The analysis included a total of 4,578 data points (six excluded due to image quality), with each point representing a deep learning-derived BAG (predicted age subtracted by chronological age) in years. The results are multiple time point data visualized using box-violin plots, where the x-axis represents the eight scanner models and the y-axis displays the corresponding median values of BAG. No significant inter-scanner differences were found.

Supplementary Figure 5 ML-1118: MRI Scanner differences and Its Influence on Brain Age Gap at all EDSS categories

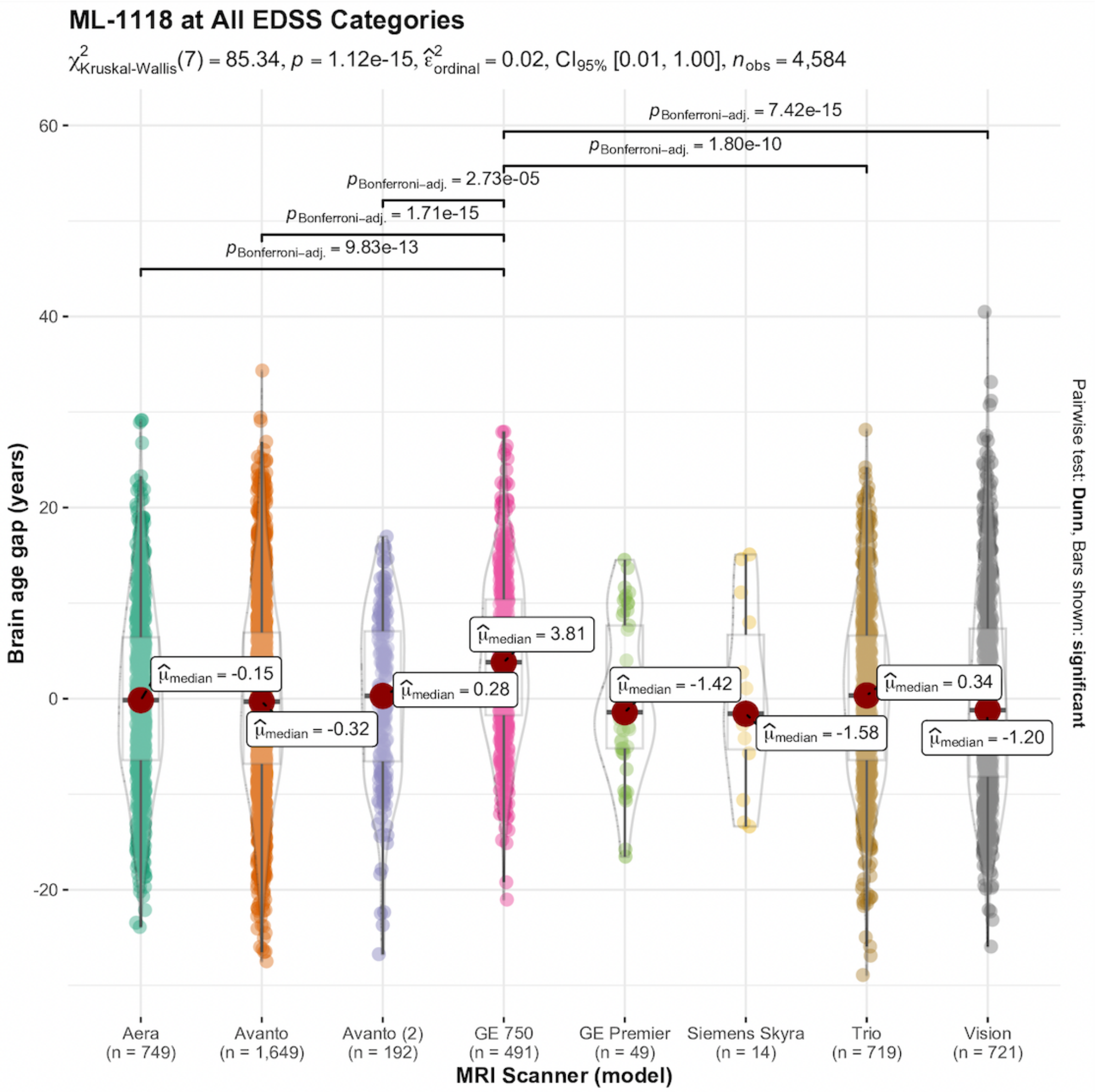

ML-1118: Machine learning 1118 features; **BAG**: Brain age gap; **EDSS**: Expanded disability status scale

The Chi-Square Kruskal-Wallis Test was used for multiple comparisons of non-parametric data with unequal variances to evaluate inter-scanner differences in ML-1118-derived BAG across all EDSS scores (range 0–9). The test revealed statistically significant overall differences between scanner models ($X^2 = 85.34$, $p < 0.001$), with a small effect size (ε^2ordinal = 0.02, 95% CI [0.01, 1.00]). The analysis included 4,584 data points, with each point representing a deep learning-derived BAG (predicted age subtracted by chronological age) in years. The results are multiple time point data visualized using box-violin plots, where the x-axis represents the eight scanner models and the y-axis displays the corresponding median values of BAG. Connecting lines indicate statistically significant pairwise differences between scanner models, with p-values (threshold = 0.05) adjusted using the Bonferroni correction for multiple comparisons.

Supplementary Figure 6 DL-SFCN: MRI Scanner differences and Its Influence on Brain Age Gap at EDSS 3.5 or less

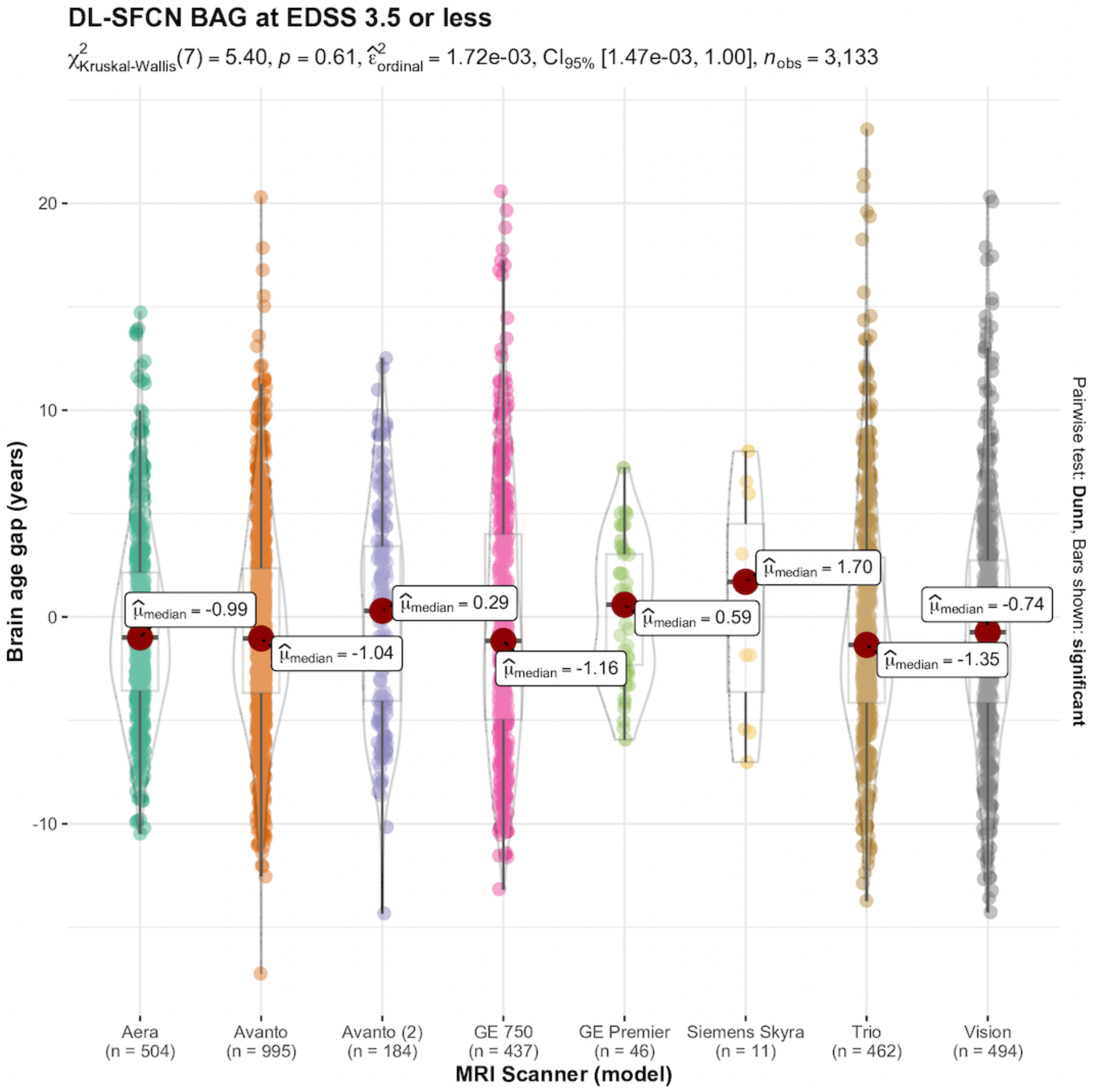

DL-SFCN: Deep-learning simple fully convolutional network; **BAG**: Brain age gap; **EDSS**: Expanded disability status scale

The Chi-Square Kruskal-Wallis Test was used for multiple comparisons of non-parametric data with unequal variances to evaluate inter-scanner differences in DL-SFCN-derived BAG at EDSS 3.5 or less (range 0–3.5). The test revealed statistically nonsignificant overall differences between scanner models ($X^2 = 5.40$, $p = 0.61$), with a small effect size (ε^2ordinal = 0.002, 95% CI [0.01, 1.00]). The analysis included a total of 3,133 data points (six excluded due to image quality), with each point representing a deep learning-derived BAG (predicted age subtracted by chronological age) in years. The results are multiple time point data visualized using box-violin plots, where the x-axis represents the eight scanner models and the y-axis displays the corresponding median values of BAG. No significant inter-scanner differences were found.

Supplementary Figure 7 ML-1118: MRI Scanner differences and Its Influence on Brain Age Gap at EDSS 3.5 or less

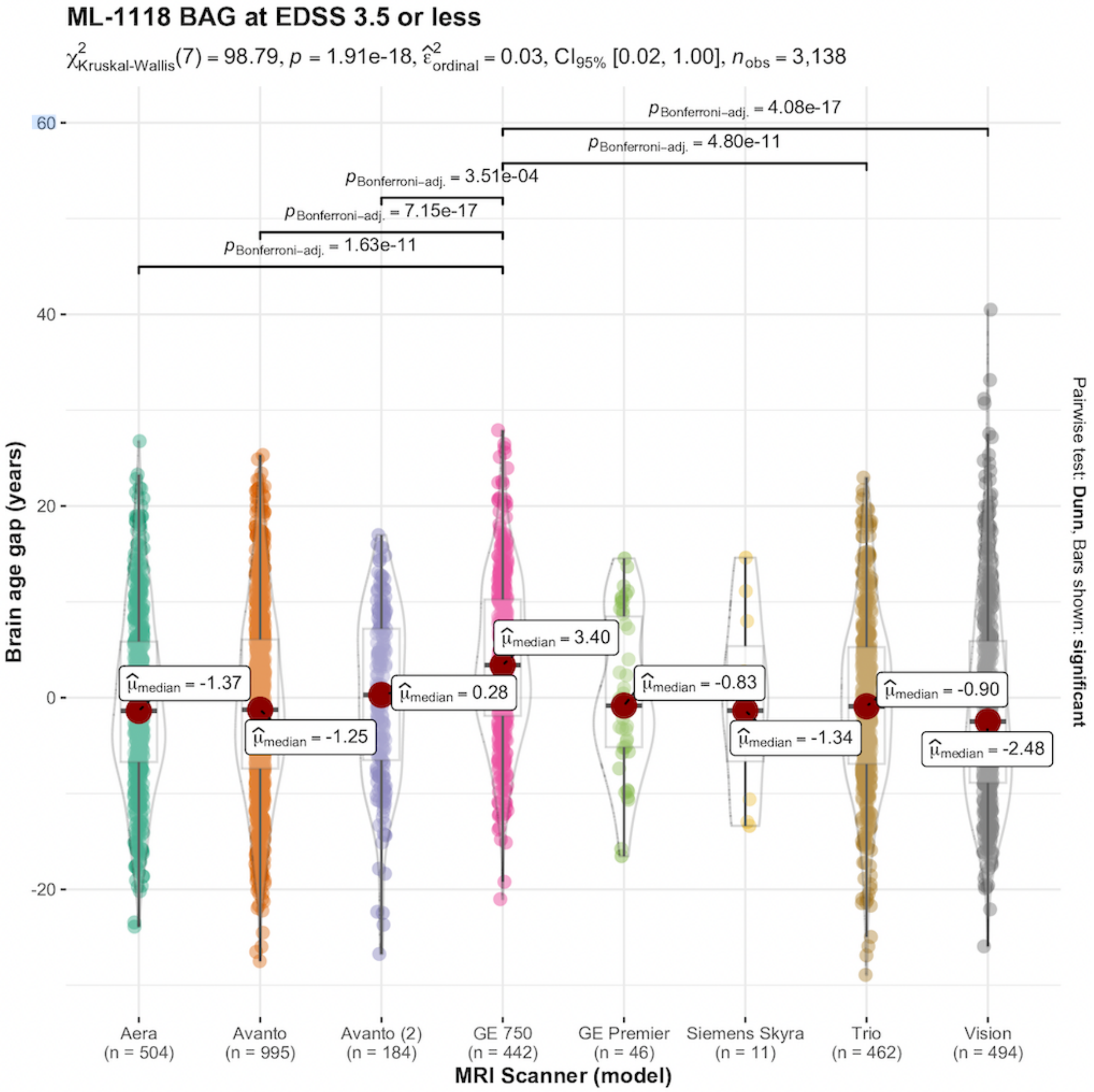

ML-1118: Machine learning 1118 features; **BAG**: Brain age gap; **EDSS**: Expanded disability status scale

The Chi-Square Kruskal-Wallis Test was used for multiple comparisons of non-parametric data with unequal variances to evaluate inter-scanner differences in ML-1118-derived BAG at EDSS 3.5 or less (range 0–3.5). The test revealed statistically significant overall differences between scanner models ($X^2 = 98.79$, $p < 0.001$), with a small effect size (ε^2ordinal = 0.03, 95% CI [0.02, 1.00]). The analysis included 3,138 data points, with each point representing a deep learning-derived BAG (predicted age subtracted by chronological age) in years. The results are multiple time point data visualized using box-violin plots, where the x-axis represents the eight scanner models and the y-axis displays the corresponding median values of BAG. Connecting lines indicate statistically significant pairwise differences between scanner models, with p-values (threshold = 0.05) adjusted using the Bonferroni correction for multiple comparisons.

Supplementary Figure 8 DL-SFCN: MRI Scanner differences and Its Influence on Brain Age Gap at EDSS 4.0 – 6.0

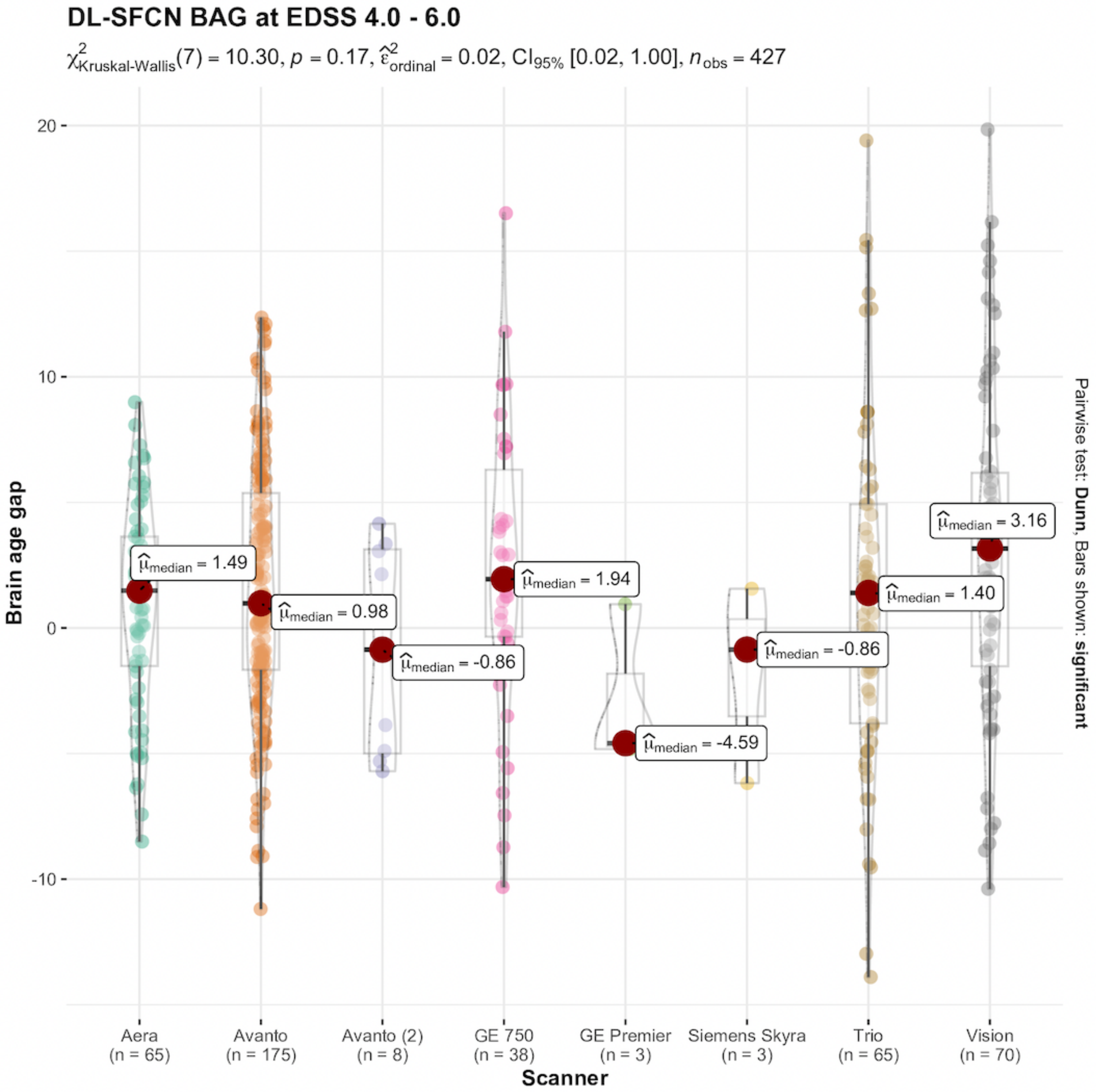

DL-SFCN: Deep-learning simple fully convolutional network; **BAG**: Brain age gap; **EDSS**: Expanded disability status scale

The Chi-Square Kruskal-Wallis Test was used for multiple comparisons of non-parametric data with unequal variances to evaluate inter-scanner differences in DL-SFCN-derived BAG at EDSS 4.0-6.0. The test revealed statistically nonsignificant overall differences between scanner models ($X^2 = 10.30$, $p = 0.17$), with a small effect size (ε^2ordinal = 0.02, 95% CI [0.02, 1.00]). The analysis included a total of 427 data points, with each point representing a deep learning-derived BAG (predicted age subtracted by chronological age) in years. The results are multiple time point data visualized using box-violin plots, where the x-axis represents the eight scanner models and the y-axis displays the corresponding median values of BAG. No significant inter-scanner differences were found.

Supplementary Figure 9 ML-1118: MRI Scanner differences and Its Influence on Brain Age Gap at EDSS 4.0 – 6.0

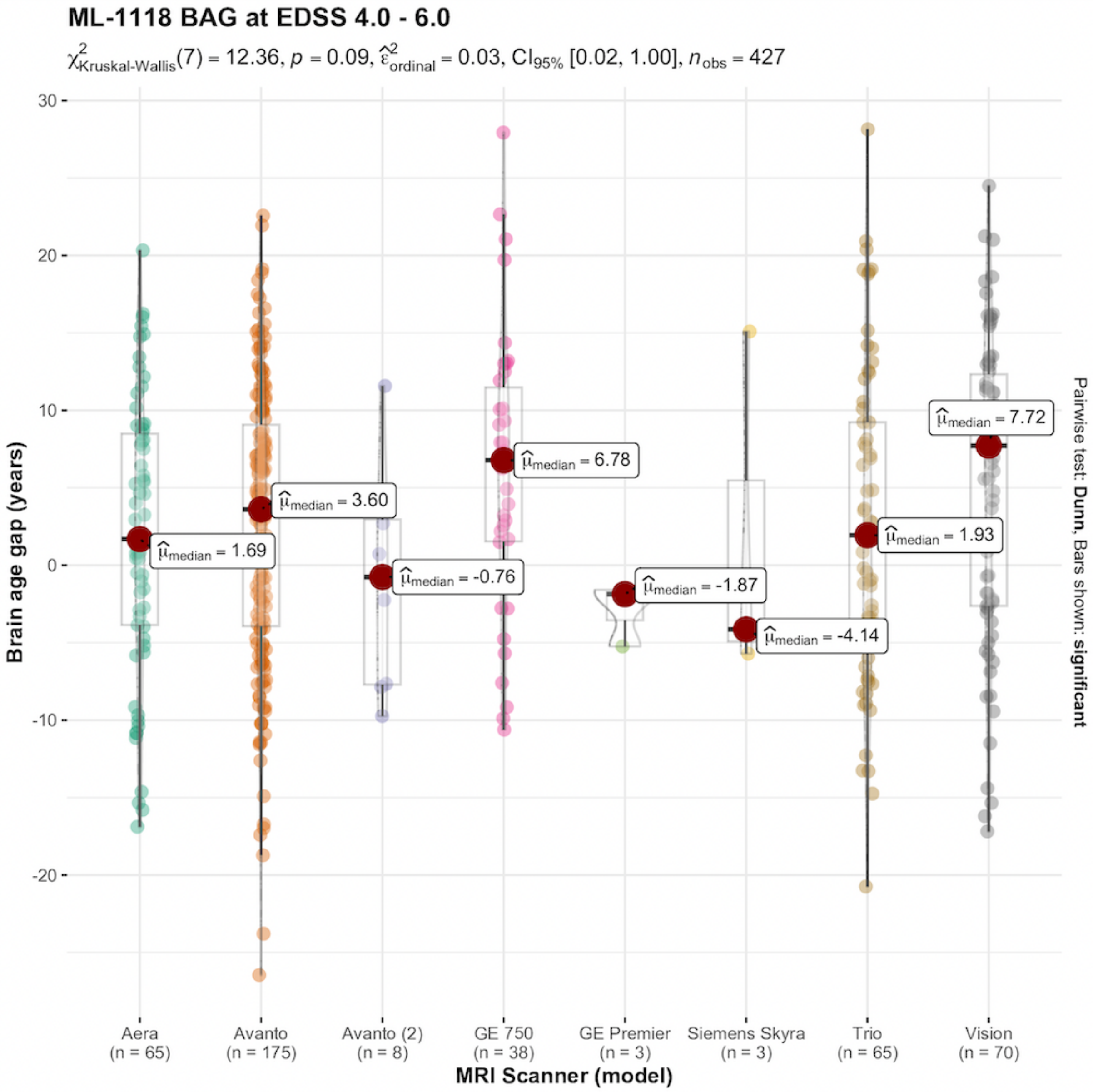

ML-1118: Machine learning 1118 features; **BAG**: Brain age gap; **EDSS**: Expanded disability status scale

The Chi-Square Kruskal-Wallis Test was used for multiple comparisons of non-parametric data with unequal variances to evaluate inter-scanner differences in ML-1118-derived BAG at EDSS 4.0-6.0. The test revealed statistically nonsignificant overall differences between scanner models ($X^2 = 12.36$, $p = 0.09$), with a small effect size (ε^2ordinal = 0.03, 95% CI [0.02, 1.00]). The analysis included 427 data points, with each point representing a deep learning-derived BAG (predicted age subtracted by chronological age) in years. The results are multiple time point data visualized using box-violin plots, where the x-axis represents the eight scanner models and the y-axis displays the corresponding median values of BAG. No significant inter-scanner differences were found.

Supplementary Figure 10 DL-SFCN: MRI Scanner Variability and Its Influence on Brain Age Gap at EDSS above 6·0

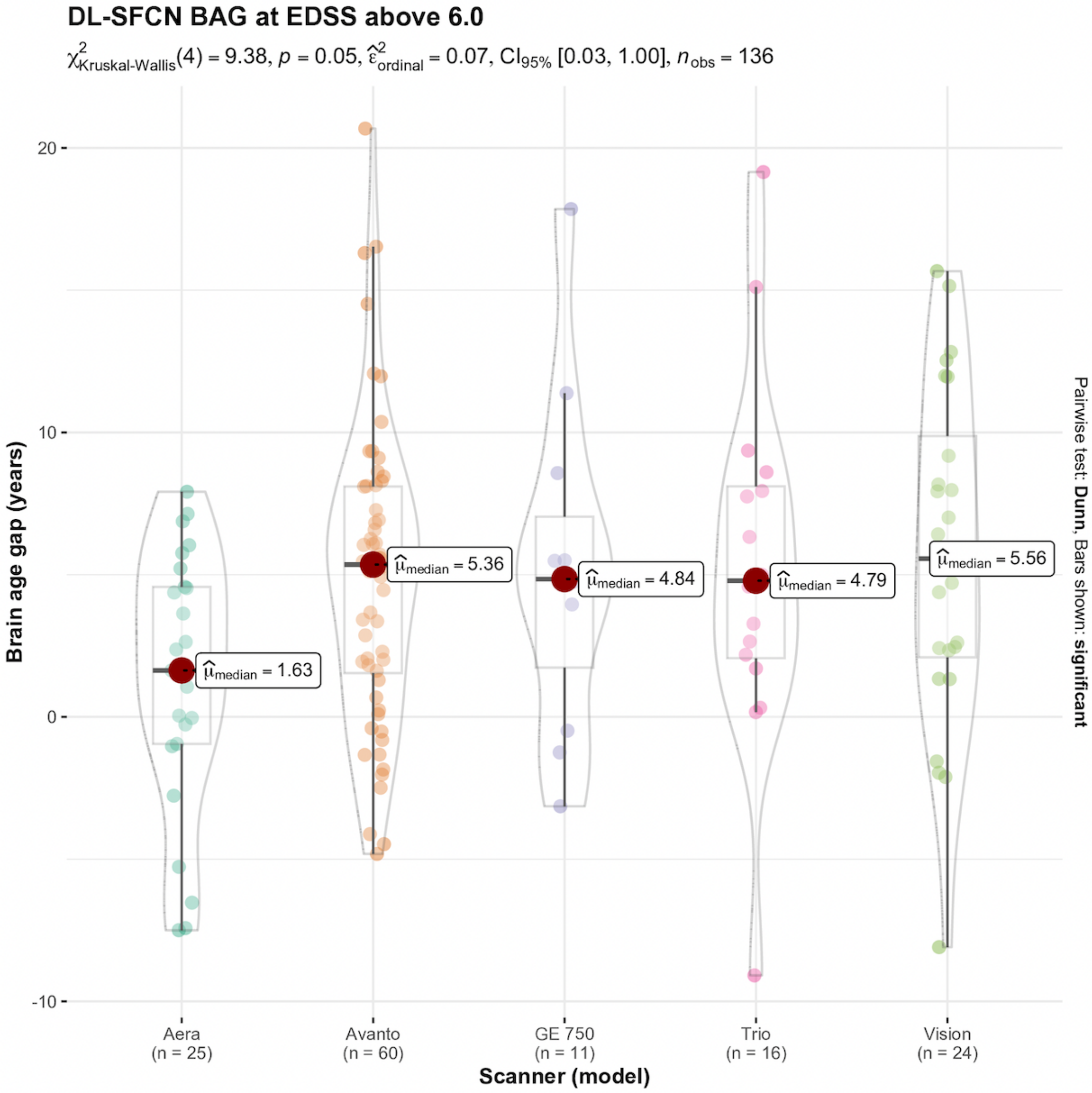

DL-SFCN: Deep-learning simple fully convolutional network; **BAG**: Brain age gap; **EDSS**: Expanded disability status scale

The Chi-Square Kruskal-Wallis Test was used for multiple comparisons of non-parametric data with unequal variances to evaluate inter-scanner differences in DL-SFCN-derived BAG at EDSS above 6.0. The test revealed statistically significant overall differences between scanner models ($X^2 = 9.38$, $p = 0.05$), with a small effect size (ε^2ordinal = 0.07, 95% CI [0.03, 1.00]). The analysis included a total of 136 data points, with each point representing a deep learning-derived BAG (predicted age subtracted by chronological age) in years. The results are multiple time point data visualized using box-violin plots, where the x-axis represents the eight scanner models and the y-axis displays the corresponding median values of BAG. No significant inter-scanner differences were found.

Supplementary Figure 11 ML-1118: MRI Scanner Variability and Its Influence on Brain Age Gap at EDSS above 6·0

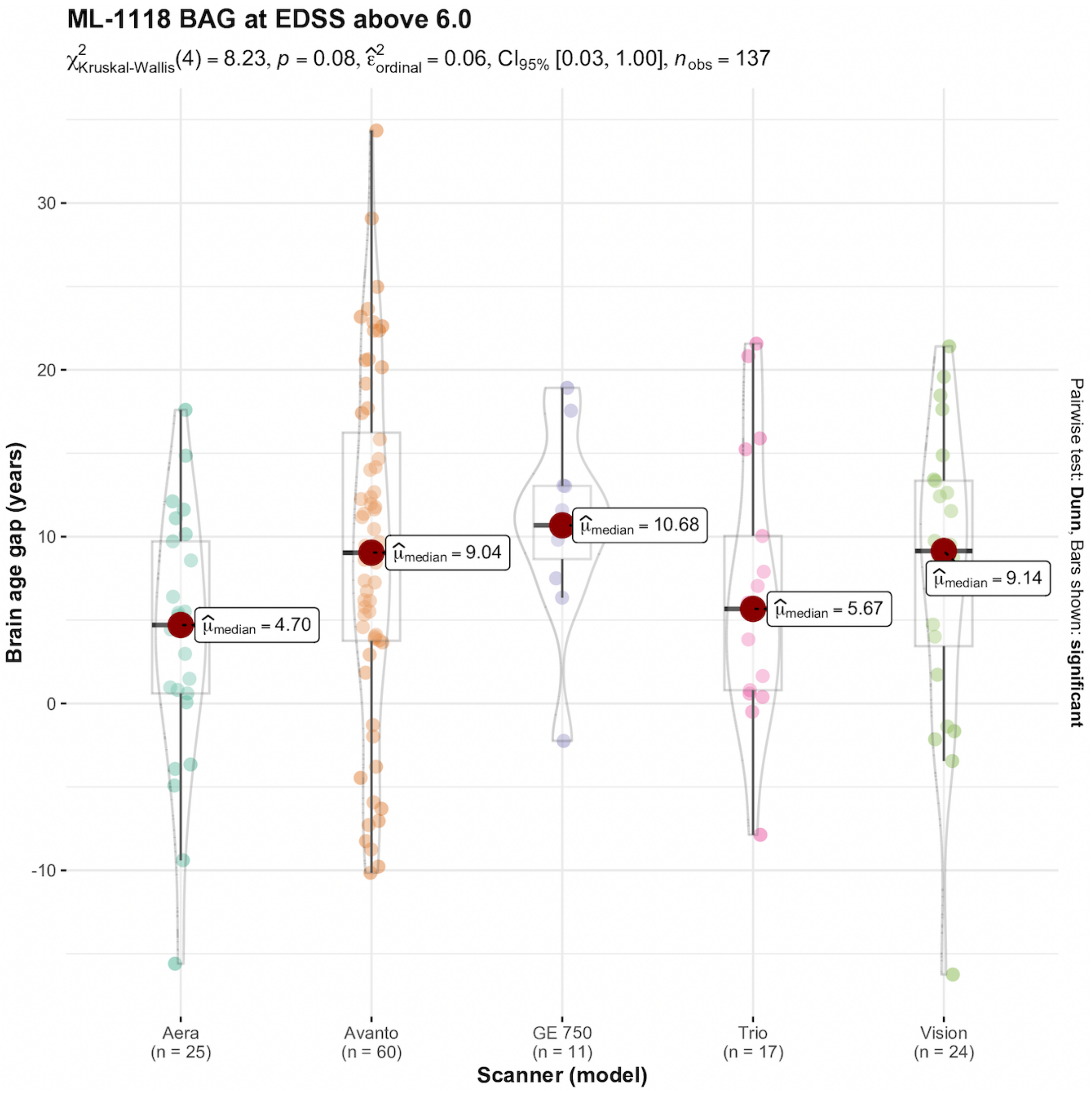

ML-1118: Machine learning 1118 features; **BAG**: Brain age gap; **EDSS**: Expanded disability status scale

The Chi-Square Kruskal-Wallis Test was used for multiple comparisons of non-parametric data with unequal variances to evaluate inter-scanner differences in ML-1118-derived BAG at EDSS above 6.0. The test revealed statistically nonsignificant overall differences between scanner models ($X^2 = 8.23$, $p = 0.08$), with a small effect size (ε^2ordinal = 0.06, 95% CI [0.03, 1.00]). The analysis included 137 data points, with each point representing a deep learning-derived BAG (predicted age subtracted by chronological age) in years. The results are multiple time point data visualized using box-violin plots, where the x-axis represents the eight scanner models and the y-axis displays the corresponding median values of BAG. No significant inter-scanner differences were found.

References:

1. Koller M. robustlmm : An *R* Package for Robust Estimation of Linear Mixed-Effects Models. *J Stat Soft*. 2016;75(6). doi:10.18637/jss.v075.i06

2. Huber PJ. Robust Estimation of a Location Parameter. *The Annals of Mathematical Statistics*. 1964;35(1):73-101. doi:10.1214/aoms/1177703732

3. Diedenhofen B, Musch J. cocor: A Comprehensive Solution for the Statistical Comparison of Correlations. *PLoS One*. 2015;10(4):e0121945. doi:10.1371/journal.pone.0121945

4. Koo TK, Li MY. A Guideline of Selecting and Reporting Intraclass Correlation Coefficients for Reliability Research. *J Chiropr Med*. 2016;15(2):155-163. doi:10.1016/j.jcm.2016.02.012
